# C-terminal long-QT type 1 R562S-Kv7.1 variant, the first variant in helix C impairing β-adrenergic response of the slow delayed rectifier K^+^ channel

**DOI:** 10.1093/europace/euag156

**Published:** 2026-06-18

**Authors:** Martin Král, Olga Švecová, Roman Kula, Nina Kadášová, Jindřich Lněnička, Iva Synková, Dominika Traj, Larisa Chmelikova, Michal Pásek, Jan Hošek, Katarzyna Anna Radaszkiewicz, Irena Andršová, Pavel Vít, Karel Berka, Tomáš Novotný, Markéta Bébarová

**Affiliations:** Department of Physiology, Faculty of Medicine, Masaryk University, Kamenice 5, Brno 625 00, Czech Republic; Department of Physiology, Faculty of Medicine, Masaryk University, Kamenice 5, Brno 625 00, Czech Republic; Department of Physiology, Faculty of Medicine, Masaryk University, Kamenice 5, Brno 625 00, Czech Republic; Department of Anaesthesiology, Resuscitation and Intensive Care, The Tomas Bata Regional Hospital, Havlíčkovo nábřeží 600, Zlín 762 75, Czech Republic; Department of Physical Chemistry, Faculty of Science, Palacký University, 17. listopadu 1192/12, Olomouc 771 46, Czech Republic; Department of Physical Chemistry, Faculty of Science, Palacký University, 17. listopadu 1192/12, Olomouc 771 46, Czech Republic; Center of Molecular Biology and Genetics, Department of Internal Medicine, Hematology and Oncology, University Hospital Brno and Faculty of Medicine, Masaryk University, Jihlavská 20, 625 00 Brno, Czech Republic; Department of Internal Medicine and Cardiology, University Hospital Brno and Faculty of Medicine, Masaryk University, Jihlavská 20, Brno 625 00, Czech Republic; Department of Biomedical Engineering, Faculty of Electrical Engineering and Communication, Brno University of Technology, Technická 10, Brno 616 00, Czech Republic; Department of Physiology, Faculty of Medicine, Masaryk University, Kamenice 5, Brno 625 00, Czech Republic; Institute of Thermomechanics, Czech Academy of Sciences, Dolejškova 5, Prague 182 00, Czech Republic; Department of Molecular Pharmacy, Faculty of Pharmacy, Masaryk University, Palackého třída 1946/1, Brno 612 00, Czech Republic; Department of Experimental Biology, Faculty of Science, Masaryk University, Kamenice 5, Brno 625 00, Czech Republic; Department of Internal Medicine and Cardiology, University Hospital Brno and Faculty of Medicine, Masaryk University, Jihlavská 20, Brno 625 00, Czech Republic; Department of Paediatrics, University Hospital Brno and Faculty of Medicine, Masaryk University, Černopolní 9, Brno 613 00, Czech Republic; Department of Physical Chemistry, Faculty of Science, Palacký University, 17. listopadu 1192/12, Olomouc 771 46, Czech Republic; Department of Internal Medicine and Cardiology, University Hospital Brno and Faculty of Medicine, Masaryk University, Jihlavská 20, Brno 625 00, Czech Republic; Department of Physiology, Faculty of Medicine, Masaryk University, Kamenice 5, Brno 625 00, Czech Republic; Department of Internal Medicine and Cardiology, University Hospital Brno and Faculty of Medicine, Masaryk University, Jihlavská 20, Brno 625 00, Czech Republic

**Keywords:** long QT, *KCNQ1*, *I*_Ks_, Genetic variant, R562S, β-adrenergic stimulation

## Abstract

**Aims:**

Kv7.1 variants, associated with long QT syndrome type 1 (LQT1) and altering the function of the slow delayed rectifier K^+^ (*I*_Ks_) channel, may result in arrhythmias, especially during exercise. This study focused on complex analysis of the R562S-Kv7.1 variant located in helix C of the Kv7.1 C terminus, which was identified in four putatively unrelated families in the Czech Republic.

**Methods and results:**

The clinical and genetic investigation was followed by functional analysis (whole-cell patch clamp, confocal microscopy, computational simulations) and structural modelling. The genetic analysis suggested that R562S-Kv7.1 might be a founder LQT1 variant in Central Europe. R562S carriers showed a significantly prolonged corrected QT (QTc) interval at rest and a significantly higher QTc prolongation after exercise vs. healthy relatives. The functional analysis of R562S channels demonstrated their preserved membrane localization, a significant decrease in *I*_Ks_ with a rightward shift of the voltage dependence of activation, and, importantly, a lack of responsiveness to β-adrenergic stimulation. The latter seems to be related to a modified interaction of the modulatory KCNE1 subunit with Kv7.1. The pro-arrhythmic potential of R562S dysfunction, mediated by delayed afterdepolarizations during β-adrenergic stimulation, could be effectively prevented by mild (5%) inhibition of L-type Ca^2+^ current (*I*_Ca_).

**Conclusion:**

R562S-Kv7.1 is the first variant in helix C causing an impaired response of *I*_Ks_ channel to β-adrenergic stimulation, likely due to altered interactions between channel subunits, namely Kv7.1 and KCNE1. Mild *I*_Ca_ inhibition was suggested as a new treatment option.

## Graphical Abstract

**Graphical Abstract:**
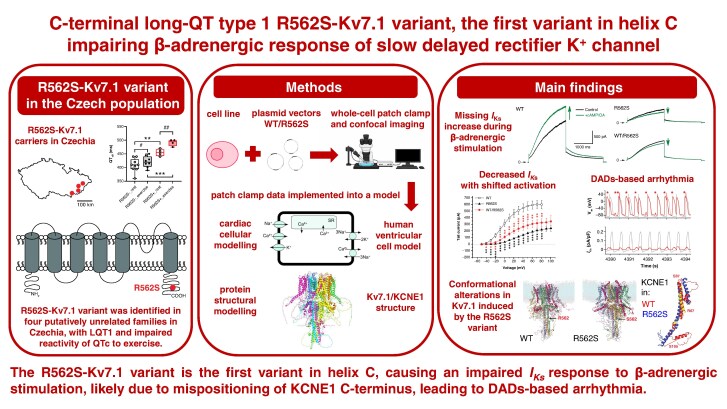

What’s new?

- A missense variant R562S in helix C of the Kv7.1 protein has been identified in four unrelated families with long QT syndrome type 1 in the Czech Republic. The founder character of the variant was suggested by haplotype analysis, but the conclusion cannot be made without broader population analysis.
- The R562S-Kv7.1 is the first variant in helix C causing an impaired response of the *I*_Ks_ channel to β-adrenergic stimulation. This corresponds to an excessive corrected QT prolongation in R562S carriers after exercise. An altered interaction between the modulatory subunit KCNE1 and the Kv7.1 channel was proposed as a mechanism for the lack of β-adrenergic response.
- Arrhythmogenesis triggered by delayed afterdepolarizations is likely and may be prevented by mild *I*_Ca_ inhibition. We suggest this as a novel therapeutic strategy in patients in whom the standard β-blocking treatment is ineffective or not well tolerated.

## Introduction

The long QT syndrome (LQTS) type 1 (LQT1) is the most often identified subtype of the syndrome, being present in 40–55% of LQTS patients.^1^ It is associated with pathogenic variants in the *KCNQ1* gene encoding the Kv7.1 protein, the α-subunit of the slow delayed rectifier K^+^ (*I*_Ks_) channel.

*I*_Ks_ plays an important role in cardiac repolarization, especially at an increased sympathetic tone. Upon activation of β-adrenergic receptors, cyclic adenosine monophosphate (cAMP)–dependent protein kinase A (PKA), bound to the anchoring protein Yotiao, phosphorylates the N terminus of Kv7.1, especially at position S27. Zhong *et al.*^2^ suggest that the resulting PKA-mediated enhancement of *I*_Ks_ depends on interactions involving the modulatory β-subunit KCNE1 and phosphatidylinositol 4,5-bisphosphate (PIP2), although the precise underlying mechanisms remain incompletely understood. These interactions are thought to promote the transition of Kv7.1 to the activated state and its stabilization. The enhanced *I*_Ks_ during β-adrenergic stimulation thus leads to accelerated repolarization and shortening of the cardiac action potential at high heart rates.^3–5^ If this essential regulation of *I*_Ks_ channel is impaired, cardiac repolarization is delayed, resulting in a prolonged QT interval corrected to the heart rate (QTc) on an electrocardiogram (ECG). This may result in life-threatening arrhythmias in the affected patients, typically during and after physical exertion.^6^

In 2012, we published a study showing a spectrum of sequence variants found in our LQT1 and LQT2 patients.^7^ Besides others, a C-terminal variant c.1686G>C (p.R562S) in the *KCNQ1* gene was identified for the first time. This variant is localized in helix C, one of four functionally important helices, namely helices A, B, C, and D, in the Kv7.1 C terminus.^8,9^ We started functional analysis of the R562S variant in 2017. In 2018, Liu *et al.*^10^ reported their data on this variant, including fundamental electrophysiological and biochemical analysis. The authors demonstrated that the R562S variant resulted in a rightward shift of the voltage dependence of steady-state activation and a lower channel affinity to PIP2. However, the functionally crucial responsiveness of the variant to β-adrenergic stimulation has not been tested.

In this study, we provide clinical, genetic, and functional characteristics of the R562S variant in the Kv7.1 protein identified in four putatively unrelated families in the Czech Republic. Besides clinical and genetic analysis, including the haplotype analysis suggesting the potential founder character of the variant, we especially investigated its impact on β-adrenergic stimulation of *I*_Ks_ channel, as well as on the related arrhythmogenesis.

## Methods

A detailed description of the methods is provided in the Supplementary material.

### Clinical diagnostics

The study conformed to the principles outlined in the Declaration of Helsinki and was approved by the Multicenter Ethical Committee, University Hospital Brno (Brno, Czech Republic). All participants signed a written informed consent form. In participants under the age of 18 years, written informed consent was obtained from a parent and/or legal guardian.

Long QT syndrome diagnosis was established according to European Society of Cardiology guidelines.^11^ All individuals underwent clinical examination and bicycle ergometry. The initial stress was set to 0.5 W/kg and increased by 0.5 W/kg every 3 min to achieve a heart rate higher than the sub-maximal value for age and sex. A 12-lead ECG with Mason–Likar modification was employed. QT and RR intervals were measured manually (the end of the QT interval was established using the threshold method); the Bazett’s correction formula was used.

### Genetic analysis

Molecular analysis of LQTS-associated genes was performed according to current practices for molecular genetic diagnostics: Sanger sequencing of three LQTS major genes on ABI 3100 Genetic Analyser (Applied Biosystems, Foster City, CA, USA) and massive parallel sequencing on MiSeq (Illumina, San Diego, CA, USA). Genetic counselling and testing of first-degree relatives were offered to patients at risk. The presumed impact of the R562S variant on *I*_Ks_ channel function was predicted using MutationTaster and FATHMM.^12^ Conservation of the impacted amino acid position was assessed using Likelihood Ratio Test and MutationAssessor. Allele frequency of the substitution was determined using online databases ExAC^13^ and GnomAD.^14^

The University of California, Santa Cruz Genome Browser was used to select 9 short tandem repeat (STR) markers spanning the ∼11.9 Mb region of chromosome 11 (including the *KCNQ1* gene) for the haplotype analysis (see Supplementary material online, *Table S1*). Multiplex polymerase chain reaction with fluorescently labelled primers and fragment analysis with capillary electrophoresis were performed on SeqStudio Genetic Analyzer (Applied Biosystems, Foster City, CA, USA) and the segregation in families was studied to identify the haplotype linked to R562S variant. Population allele frequency analysis was performed by capillary electrophoresis after identifying a common STR allele at marker D11S4088 in all variant carriers (the control group: 52 unrelated patients).

### Functional analysis

Plasmids containing wild-type (WT) human *KCNQ1* in a pIRES2-eGFP vector, *KCNE1* in a pKB-CMV vector, and *Yotiao* in a pGW1 vector were isolated from *Escherichia coli* using the endotoxin-free QIAprep Spin Miniprep Kit (QIAGEN, Hilden, Germany). The variant c.1686G > C in the human *KCNQ1* (p.R562S) was generated by site-directed mutagenesis using QuikChange II XL Site-Directed Mutagenesis Kit (Agilent Technologies, Cedar Creek, TX, USA).

TransFast Transfection Reagent (Promega, Madison, WI, USA) was used for transfection of the plasmids *KCNQ1*, *KCNE1*, and *Yotiao* (the molar ratio 1:2:4, total amount of *KCNQ1* DNA 1 µg, ratio of DNA to transfection agent 1:1.5) into Chinese hamster ovary (CHO) cells cultured at 37°C/5% CO_2_ in the Ham’s F-12 medium supplemented with 10% foetal calf serum, 2 mM glutamine, and 0.005% gentamycin. *KCNQ1* was transfected in one of three ways: (i) WT alone (1 µg); (ii) R562S alone (1 µg); and (iii) WT and R562S in the ratio 1:1 (0.5 µg of WT and 0.5 µg of R562S variant; WT/R562S) to mimic the heterozygous state in the carriers.

Biophysical analysis was performed ∼24 h after the transfection by the whole-cell patch clamp technique at 37°C using the Axopatch 200B amplifier, Digidata 1440A, and pCLAMP 9.2 software (Molecular Devices, Sunnyvale, CA, USA). The resistance of filled borosilicate glass electrodes pulled and heat-polished using a programmable horizontal puller (Zeitz-Instrumente Vertriebs GmbH, Martinsried, Germany) was kept below 2.5 MΩ. The series resistance was compensated up to 60%. Tyrode solution of the following composition was used (in mmol/L): NaCl 132, KCl 4.8, CaCl_2_ 1.8, MgCl_2_ 1.2, 4-(2-Hydroxyethyl)piperazine-1-ethanesulfonic acid (HEPES) 10, glucose 5 (pH 7.4, NaOH). The patch electrode filling solution contained (in mmol/L): K-aspartate 110, K_2_ATP 5, CaCl_2_ 1, MgCl_2_ 1, Ethyleneglycol- bis(β-aminoethyl)-N,N,Nʹ,Nʹ-tetraacetic acid 11, HEPES 10 (pH 7.3, KOH). To simulate β-adrenergic stimulation, the pipette solution was supplemented with cAMP (200 µmol/L) and okadaic acid (OA, 0.2 µmol/L). The junction potential was ∼15 mV (calculated using Clampex).

The frequency distribution was normal in the case of the tail current magnitude (not illustrated), but not in the case of the cell membrane capacitance (see Supplementary material online, *Figure S1*). Considering missing proportionality between the tail current and cell membrane capacitance (see Supplementary material online, *Figure S2*), conversion of the magnitude of the current to the current density was avoided, as recommended by Kula *et al.*^15^ The average cell membrane capacitance was comparable in cells expressing WT, R562S, and WT/R562S (considering the non-normal distribution and the paper by Kula *et al.*,^16^ the geometric mean */ the geometric standard deviation (GSD) are listed: 13.5*/1.4, 13.7*/1.6, and 12.9*/1.6 pF, respectively, *n* = 21, 28, and 20; *P* > 0.05, Kruskal–Wallis test with the Dunn’s post-test).

For the fluorescence image acquisition, a confocal laser scanning microscope Leica TCS SP8 X (Leica Microsystems, Wetzlar, Germany) was used. WT and R562S human *KCNQ1* tagged with green fluorescent protein (GFP) at the 3’ terminus were transfected into CHO cells seeded on glass-bottom dishes (Cellvis, Mountain View, CA, USA) coated with fibronectin ∼48 h before the evaluation (*KCNQ1* and *KCNE1* in the molar ratio 1:2).

Unless otherwise indicated, the chemicals were purchased from Sigma-Aldrich (Prague, Czech Republic).

### Molecular dynamics simulations

To evaluate the channel's dynamic behaviour, molecular dynamics (MD) simulations were performed. The input structures for the channel tetramer were taken from the AlphaFold 3 model (for details, see the Supplementary material and Supplementary material online, *Figure S7*). All simulations were carried out using CHARMM36m^17^ force field and GROMACS 2024.3^18^ simulation package on a LUMI supercomputer and on AMD Instinct MI250X GPUs.

Production simulations were carried out for 1 µs. Systems were simulated with a 2 fs time step, under constant pressure and temperature. Hydrogen mass repartitioning was not employed. A V-rescale thermostat was used for temperature control, set to 303.15 K. A C-rescale barostat was used for pressure coupling in a semi-isotropic setting, with a reference pressure of 1 bar. Electrostatics were computed using the particle mesh Ewald method.

### Mathematical modelling in a human ventricular cell model

A previously published model of human ventricular subepicardial myocyte (the MATLAB code at: https://www.it.cas.cz/en/d3/l033/)^19^ was used to simulate the effect of the WT/R562S variant of *I*_Ks_ channels on arrhythmogenesis under β-adrenergic stimulation (1 µM isoproterenol, as described and validated in the supplementary material of Synková *et al.*^19^). In the model, the steady-state activation of *I*_Ks_ was reformulated according to new experimental data. To incorporate the experimentally observed impact of the WT/R562S variant, we reduced the maximum conductance g_Ks_ by 50% and shifted the steady-state activation to the right (see ‘R562S Variant Caused Loss of I_Ks_ Channel Function’).

The numerical solution was performed in MATLAB version 7.2 (MathWorks, Natick, MA, USA) using the built-in stiff ordinary differential equation solver (ODE-15 s). Unless otherwise stated, ionic concentrations in the extracellular bulk space [Na^+^]_b_, [K^+^]_b_, and [Ca^2+^]_b_ were set to 140, 5.4, and 1.8 mM, respectively.

### Statistical analysis

The basic evaluation and curve fitting were performed using the software Origin, version 2022b (OriginLab Corporation). The data are mostly presented by the arithmetic mean (±standard deviation from *n* patients, or ±standard error of the mean from *n* cells) except for the cell membrane capacitance and differences in relative fluorescence intensity (*Figure 2*), where the geometric mean */GSD was used because they were not normally distributed (Shapiro–Wilk test).^16^ Based on the frequency distribution of the data, non-parametric or parametric tests were used (see figure legends) to consider the statistical significance (*P* < 0.05; GraphPad Prism, version 9.5.1, GraphPad Software, Inc.).

## Results

### Families with R562S carriers—clinical and genetic profile

**Figure 1:**
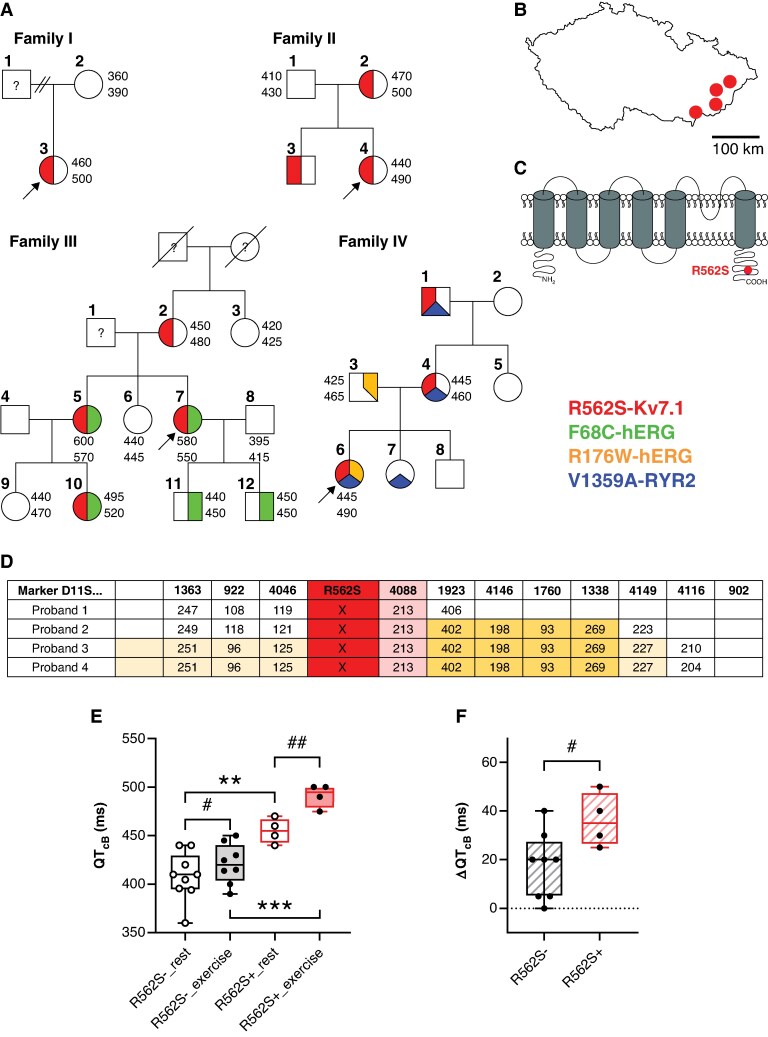
Clinical and genetic characteristics of R562S carriers. (*A*) Pedigrees of the four putatively unrelated families with corrected QT (QTc) values at rest/after exercise, where available; red—R562S-Kv7.1, green—F68C-hERG, orange—R176W-hERG, blue—V1359A-RYR2. (*B*) Residence of the four probands (circles). (*C*) Scheme of the Kv7.1 subunit with a red circle marker showing the approximate location of the R562S variant. (*D*) Haplotype analysis of the probands. (*E*) QT interval corrected to the heart rate using the Bazett’s formula (QT_cB_) at rest (empty symbols) and at the fourth minute of recovery after the exercise test (full symbols) in healthy individuals (R562S−; *n* = 8) and carriers of R562S variant (R562S+; *n* = 4); ** and *** - tatistical significance R562S− vs. R562S+ at *P* < 0.01 and 0.001, respectively [one-way analysis of variance (ANOVA) with the Bonferroni post-test]; ^#^ and ^##^Statistical significance of R562S−_rest vs. R562S−_exercise at *P* < 0.05, and R562S+_rest vs. R562S+_exercise at *P* < 0.01 (paired *t*-test). (*F*) Change in QT_cB_ (ΔQT_cB_) after the exercise test in R562S− and R562S+ (^#^*P* < 0.05, unpaired *t*-test).

The same substitution c.1686G>C in the *KCNQ1* gene has been identified in four putatively unrelated families in the Czech Republic (*Figure* *1A* and *B*). This substitution resulted in an exchange of the basic amino acid arginine, conserved at this position across various species, to the hydrophilic amino acid serine in the C-terminal region of the Kv7.1 protein (p.R562S; *Figure 1C*). The predictive tools suggested a damaging or disease-causing effect of the R562S variant (MutationTaster: score 110, predicted as disease-causing; FATHMM: −7.47, predicted as damaging).

*Table 1* summarizes the genetic and clinical characteristics of the patient cohort, and representative ECG recordings are shown in Supplementary material online, *Figures S4*–*S6*. We have identified five pure heterozygous R562S carriers across these four families, most of them in Families I and II (*Figure 1A*; QTc values for all investigated family members are presented in the pedigrees). In Family I, a single heterozygous R562S carrier, the proband (I:3; see Supplementary material online, *Figure S4*), was diagnosed after an incidental finding of a prolonged QT interval on ECG without experiencing any symptoms. The QTc of 460 ms at rest was increased to 500 ms in the fourth minute of post-exercise recovery (no arrhythmias during the exercise test), in contrast to 360 and 390 ms, respectively, in her healthy mother (I:2). In Family II, three heterozygous R562S carriers have been found. Unfortunately, only two of them agreed with a detailed clinical investigation. These two investigated carriers (female proband, II:4, and her mother, II:2) had QTc of 440 and 470 ms at rest and 490 and 500 ms in the fourth minute of post-exercise recovery, both without arrhythmia during the exercise test. The female proband (II:4) presented with intermittent episodes of chest pain, predominantly occurring after physical exertion such as running. These episodes were occasionally accompanied by palpitations and typically resolved within 5 min of cessation of activity or assuming a recumbent position. The proband's mother (II:2) reported a history of presyncope following physical exercise and recurrent episodes of dizziness before the diagnosis.

**Table 1:** Clinical and genetic characteristics of the patient cohort

Patient No.	Kv7.1 variant	Other known variants	QTc at rest (ms)	QTc after exercise (ms)	Arrhythmia(s) during exercise test	Symptoms
I:2	WT	None	360	390	None	None
I:3	R562S	None	460	500	None	None
II:1	WT	None	410	430	None	None
II:2	R562S	None	470	500	None	Presyncope during exercise, dizziness
II:3	R562S	None	x	x	X	x
II:4	R562S	None	440	490	None	Chest pain after exertion, palpitations
III:2	R562S	None	450	480	None	Intermittent palpitations
III:3	WT	None	420	425	None	None
III:5	R562S	F68C-KCNH2	600	570	None	Presyncope
III:6	WT	None	440	445	None	None
III:7	R562S	F68C-KCNH2	580	550	None	Recurrent syncope
III:8	WT	None	395	415	None	None
III:9	WT	None	440	470	None	None
III:10	R562S	F68C-KCNH2	495	520	None	None
III:11	WT	F68C-KCNH2	440	450	None	None
III:12	WT	F68C-KCNH2	450	450	None	None
IV:1	R562S	V1359A-RYR2	x	x	x	x
IV:2	WT	None	x	x	x	x
IV:3	WT	R176W-KCNH2	425	465	None	None
IV:4	R562S	V1359A-RYR2	445	460	None	Syncope
IV:5	WT	None	x	x	x	x
IV:6	R562S	R176W-KCNH2 V1359A-RYR2	445	490	None	Syncope
IV:7	WT	V1359A-RYR2	x	x	x	x
IV:8	WT	none	x	x	x	x

x: data not available (refused clinical examination).

QTc, corrected QT; WT, wild-type.

Most of the heterozygous R562S carriers from Families III and IV also carry another variant or even two other variants in different genes (*Figure 1A*). The only pure heterozygous R562S carrier from Family III (III:2; see Supplementary material online, *Figure S5*) had QTc 450 ms at rest and 480 ms in the fourth minute of post-exercise recovery. This female patient reported intermittent episodes of palpitations. In the proband (III:7, the daughter of the above-mentioned pure R562S carrier) and in the other four R562S carriers from Family III (III:5 and III:10), an additional variant c.203T>G in the *KCNH2* gene (p.F68C) was found, originally assessed as a variant of uncertain significance (VUS) according to available guidelines and database (ClinVar). In contrast, O'Neill *et al.*^20^ have recently reported a severe trafficking defect in the F68C variant, which may thus contribute to the observed phenotype. However, normal-to-borderline QTc interval durations were observed in pure heterozygous F68C carriers both at rest and after exercise (see III:11 and III:12 in *Figure 1A*). This suggests limited pathogenic potential of the F68C variant, at least when present alone. In contrast, its impact may be significantly increased, when present together with the R562S variant, as was demonstrated by an extreme QTc prolongation in patients carrying both these variants (III:5, III:7, and III:10) in comparison with the pure R562S carrier in this family (III:2). An extremely long QTc of 580 ms at rest and 550 ms in the fourth minute of post-exercise recovery was documented in the case of the proband (III:7). Due to recurrent syncope despite the maximum tolerated dose of β-blockers, the proband underwent implantation of an implantable cardioverter-defibrillator. The proband’s sister, carrying the same variants (R562S-Kv7.1 and F68C-hERG; III:5), showed a notched T-wave morphology (T2 peak higher than T1 peak) and QTc of 600 and 570 ms at rest and after exercise, respectively.

The proband from Family IV (IV:6; see Supplementary material online, *Figure S6*) carries three various variants, namely our target R562S variant, and variants in the *KCNH2* (c.526C>T, p.R176W) and *RYR2* (c.4076T>C, p.V1359A) genes. The first one, R176W, was previously identified as a Finnish founder variant.^21,22^ Its loss-of-function and pro-arrhythmic potential were documented by Lahti *et al.*^23^ and decreased channel trafficking to 20% by O'Neill *et al.*^20^ Hence, this variant may indeed contribute to the clinical phenotype of the proband carrying both R562S-Kv7.1 and R176W-hERG variants (IV:6). Interestingly, the R176W-hERG variant did not result in QTc prolongation in the pure R176W carrier, the father of the proband (IV:3). The V1359A-RYR2 variant was also predicted as a VUS, and we have not found any study analysing its functional impact. The proband (IV:6) experienced syncope at the age of 6; the QTc interval was 445 ms at rest and 490 ms in the fourth minute of post-exercise recovery. Despite carrying the RYR2 variant, no adrenergic-dependent ventricular arrhythmias have been observed both in the proband (IV:6) and in her mother, also carrying the V1359A-RYR2 variant (IV:4), during repeated exercise tests (family members IV:1 and IV:7, who also carry the V1359A-RYR2 variant, did not agree with the clinical examination). Hence, the pathogenic potential of the V1359A-RYR2 variant is likely limited. In this family, no pure R562S carrier has been identified so far. The proband’s places of residence are shown in *Figure 1B*.

The haplotype analysis of all four probands revealed a common haplotype in the near locus D11S4088 (the pink table column on the right side of R562S in *Figure 1D*). This suggests that they are likely related, i.e. having a common ancestor in the past. This marker was highly polymorphic in the control population; the fragment lengths varied between 203 and 253 bp.^19^ Considering our previous analysis,^19^ the probability of identifying the same haplotype in four random unrelated families is 0.125^4^ = 0.000244. Thus, the founder character of the R562S-Kv7.1 variant in our population is possible. However, no conclusion could be drawn. It would require a more extensive population analysis, which is not available, because a limited number of R562S carriers have been identified in our population so far.

We observed a significantly longer QTc interval duration in R562S carriers in comparison with healthy relatives, both at rest and during recovery after the exercise test (*Figure 1E*). The prolongation of the QTc interval in R562S carriers after exercise was significantly greater than in their healthy relatives (*P* < 0.05, *Figure 1F*).

### Subcellular localization of homozygous and heterozygous R562S channels

**Figure 2:**
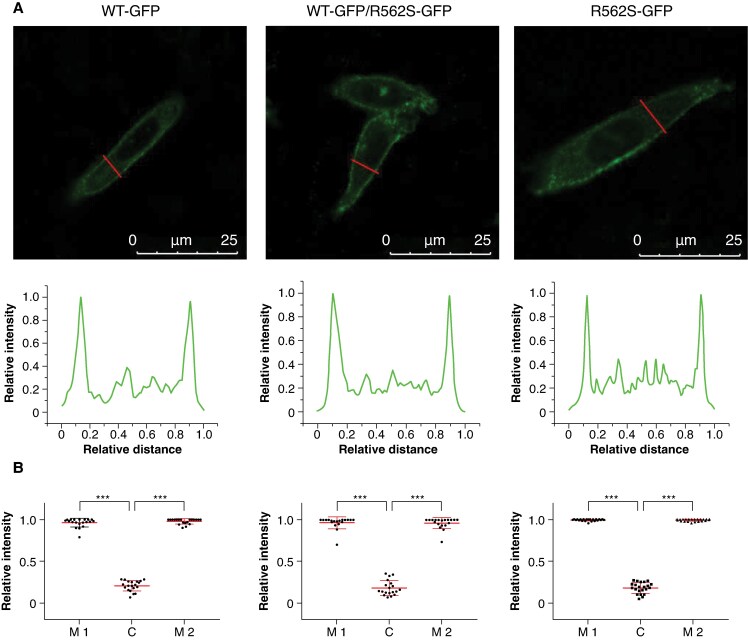
Subcellular localization of wild-type (WT)- and R562S-Kv7.1 subunits (KCNE1 subunit cotransfected). (*A*) Confocal microscopic images of representative Chinese hamster ovary (CHO) cells expressing WT-green fluorescent protein (GFP) (i.e. tagged with GFP at the C-terminal end), R562S-GFP, or both WT-GFP and R562S-GFP subunits (upper panels), and respective relative fluorescence intensity profiles (lower panels) under the lines crossing the cells in the upper panels; scale bar, 25 µm. (*B*) The resulting maximal relative fluorescence intensity at the cell membrane (M1 and M2) and its mean value in the cytosol (C); in all investigated cells (*n* = 20, 19, and 22 in WT-GFP, R562S-GFP, and WT-GFP/R562S-GFP subunits, respectively; cells from 2 to 4 transfections used in each variant); ***Statistical significance of the difference in fluorescence intensity among M1, M2, and C in the respective group of cells at *P* < 0.001 (Friedman test with the Dunn’s post-test).

*Figure 2A* illustrates confocal images (upper panels) and respective relative fluorescence intensity profiles (lower panels) of representative CHO cells expressing either WT channels (WT-GFP), or homo- and heterozygous R562S channels (R562S-GFP and WT-GFP/R562S-GFP, respectively; KCNE1 was always cotransfected). The fluorescence signal was preferentially localized at the cell membrane in all cases, as also visible from the overview of all tested cells within the cell groups (*Figure 2B*; fluorescence intensity on the cell membrane, marked as M1 and M2 in x-axes of the graphs in *Figure 2B*, was significantly higher than that in the cytosol, C; *P* < 0.001). The mean values of fluorescence intensity in the cytosol did not differ among the cell groups. Thus, the R562S variant does not seem to impair the trafficking of *I*_Ks_ channels.

### R562S variant caused loss of *I*_Ks_ channel function

All functional data were measured in the presence of KCNE1 subunits. If not otherwise stated, the stimulation frequency was 0.08 Hz. The conversion of the ionic current magnitude to the current density was avoided, except for Supplementary material online, *Figure S3* (for details, see Methods and Supplementary material).

**Figure 3:**
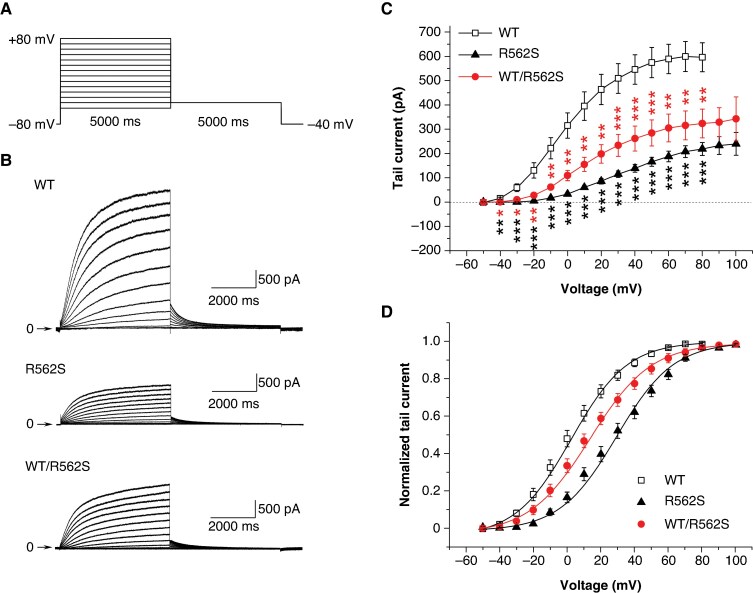
Basic biophysical characteristics of R562S channels—comparison of the homo- and heterozygous R562S channels [R562S and wild-type (WT)/R562S, respectively] vs. WT channels. (*A*) Scheme of the experimental protocol; in R562S and WT/R562S channels, the current was stimulated even up to +100 mV. (*B*) Representative recordings in all cell groups. (*C*) Average current–voltage relationship; *n* = 16, 15, and 10 in cells expressing WT (empty squares), R562S (full black triangles), and WT/R562S (full red circles) channels, respectively; ** and ***Statistical significance at *P* < 0.01 and 0.001, respectively [one-way analysis of variance (ANOVA) with the Bonferroni post-test]. (*D*) Average voltage dependence of the steady-state activation of the current [in the same cells as in (*C*)]; the data points were fitted using the Boltzmann equation with the resulting values of the half-maximal activation voltage *V*_1/2_ of +3.4 ± 2.8, +29.3 ± 2.8, and +15.1 ± 3.0 mV in WT, R562S, and WT/R562S channels, respectively (the slope factors: 14.5 ± 0.5, 17.0 ± 0.8, and 17.7 ± 1.1).

Experimental protocol and representative current traces in the human WT, R562S, and WT/R562S channels expressed in CHO cells are shown in *Figure* *3A* and *B*, respectively. WT channels were activated to their maximum tail current at voltage steps up to +80 mV, whereas the tail current in R562S channels showed a persistent rising tendency even at +80 mV. Hence, voltage steps up to +100 mV were used in R562S and WT/R562S channels (*Figure 3C*).

The homozygous R562S channels showed a typical slowly activated outward current, however, the magnitude of the tail current was significantly reduced in comparison with WT channels (e.g. at the physiologically relevant voltage of 0 mV, from 315.9 ± 52.1 pA in WT channels to 34.6 ± 6.5 pA in R562S channels, *n* = 16 and 15, respectively, *P* < 0.001; *Figure 3C*). A significant rightward shift of the voltage dependence of steady-state activation by about 26 mV was apparent in R562S channels (the half-maximal activation voltage *V*_1/2_ was +29.3 ± 2.8 vs. + 3.4 ± 2.8 mV in WT channels, *P* < 0.001; *Figure 3D*). The slope factor was also significantly different in R562S and WT channels (17.0 ± 0.8 and 14.5 ± 0.5, respectively, *P* < 0.05).

Similar, but slighter changes were apparent in WT/R562S channels (the tail current at 0 mV was 110.0 ± 20.8 pA, *P* < 0.01 vs. WT; *V*_1/2_ was +15.1 ± 3.0 mV and the slope factor 17.7 ± 1.1, *P* < 0.05 vs. WT, *n* = 10; *Figure* *3C* and *D*).

### R562S variant is the first variant in helix C of the Kv7.1 protein altering its reactivity to β-adrenergic stimulation

**Figure 4:**
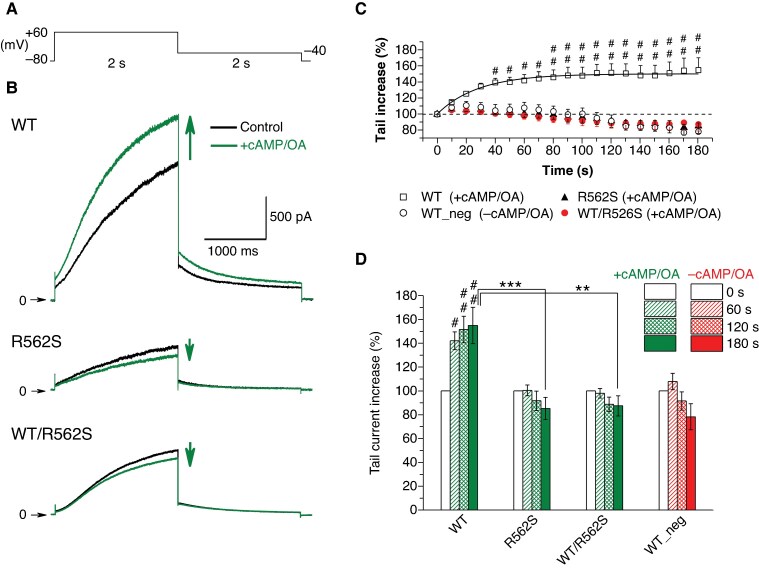
R562S-Kv7.1 variant impairs reactivity of *I*_Ks_ channel to β-adrenergic stimulation [simulated by diffusion of cyclic adenosine monophosphate, cyclic adenosine monophosphate (cAMP), and okadaic acid (OA) present in the pipette]. (*A*) Experimental protocol. (*B*) Representative traces in wild-type (WT), R562S, and WT/R562S channels after the patch rupture (at 0 s; control) and at 180 s diffusion of cAMP/OA into the measured cell (+cAMP/OA). (*C*) Time course of the average relative increase of *I*_Ks_ in WT channels (empty squares) and absent reaction in R562S (full black triangles) and WT/R562S (full red circles) channels in the presence of cAMP/OA; WT without cAMP/OA in the pipette (WT_neg; empty circles) was used as the negative control (*n* = 12, 14, 10, and 9 in WT, R562S, WT/R562S, and WT_neg, respectively); ^#^ and ^##^ - statistically significant increase of *I*_Ks_ in WT channels during 180 s diffusion of cAMP/OA vs. the control *I*_Ks_ in WT channels (at 0 s) at *P* < 0.05 and 0.01, respectively [repeated measures analysis of variance (ANOVA) with the Bonferroni post-test]. (*D*) Average relative *I*_Ks_ at 0, 60, 120, and 180 s in all tested cell groups; ** and ***Statistically significant difference between the steady-state cAMP/OA stimulation in WT vs. in R562S and WT/R562S channels at *P* < 0.01 and 0.001, respectively (one-way ANOVA with the Bonferroni post-test).

Subsequently, we focused on the reactivity of R562S channels on β-adrenergic stimulation, which was performed using supplementation of the pipette solution with 200 µmol/L cAMP and 0.2 µmol/L OA (+cAMP/OA), as in our previously published paper^19^ and according to other studies.^5,24,25^ As expected, WT current was significantly increased during the gradual diffusion of cAMP and OA into the cytosol (*Figure 4B*; the experimental protocol shown in *Figure 4A*), reaching 154.8 ± 15.3% of the control current (i.e. current at the beginning of recording) within the first 180 s of the recording (*n* = 12; *Figure* *4C* and *D*). In contrast, no increase was apparent in R562S and WT/R562S currents (*Figure 4B*). These currents even showed a gradual insignificant run-down to 85.3 ± 9.2% and 87.5 ± 8.4% of the control current at 180 s after the diffusion start (*n* = 14 and 10, respectively; at 180 s: *P* < 0.001 and 0.01 in R562S and WT/R562S, respectively, vs. WT channels; *Figure* *4C* and *D*). A similar gradual run-down was also observed in WT currents when no cAMP and OA were present in the pipette (WT_neg, -AMP/OA; the current decreased to 78.4 ± 10.8% of the control current, *n* = 9, *P* > 0.05 vs. both R562S and WT/R562S; *Figure* *4C* and *D*).

To sum up, *I*_Ks_ current in both R562S and WT/R562S channels was activated at higher membrane voltages and was decreased to <50% of the current in WT channels at physiologically relevant voltages. Importantly, the reactivity to β-adrenergic stimulation was altered by the R562S variant, further aggravating the functional defect of the channels.

### Changes in the Kv7.1-R562S structure as predicted by in silico modelling

**Figure 5:**
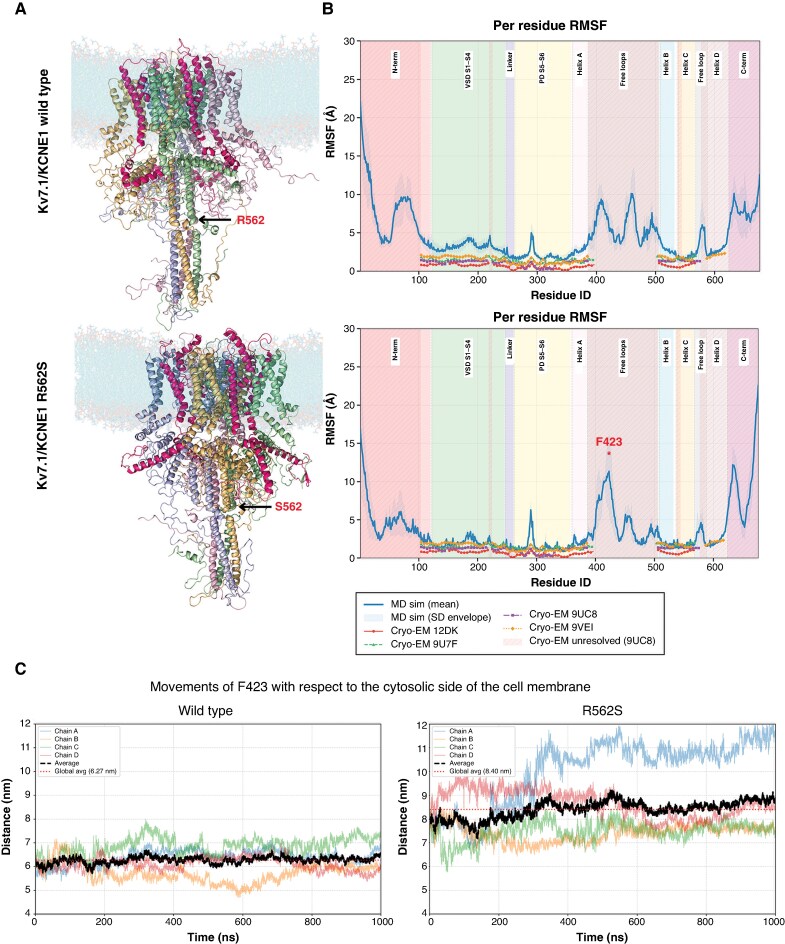
Comparison of the Kv7.1 structure between wild type (WT) and R562S as predicted by in silico structural modelling using AlphaFold 3 followed by 1-µs molecular dynamics (MD) simulations. (*A*) The predicted structures of the WT and R562S Kv7.1/KCNE1 complex in the tetrameric assembly after the MD simulations revealed structural reorganization. The black arrow shows the position of the R/S562 amino acid. (*B*) Per residue, root-mean-square fluctuation (RMSF) graphs of WT and R562S variants from the MD simulations [compared with available cryogenic electron microscopy (cryo-EM) structures] show that most of the variation is located in the free loops’ region. The asterisk is pointing out residue F423 of free loops with the most prominent rise of flexibility. (*C*) Distances of centres-of-mass of F423 in the free-loop regions from the cell membrane in individual chains in WT and R562S. Compared with the WT, the F423 in R562S is not only further from the cell membrane but also shows more flexibility, in line with RMSF plots.

Structural modelling with the subsequent MD simulations of the WT and R562S Kv7.1/KCNE1 channels (in the absence of bound ligands and calmodulin) revealed important conformational differences between the two variants (*Figure 5A*). Based on the root-mean-square fluctuation (RMSF) analysis, we observe an increase in flexibility at the beginning of the free loops that bridge helices A and B in R562S, with fluctuation amplitudes reaching 2.0 Å and notable inter-subunit variability (*Figure 5B*). Although boundaries of the loops remain unchanged, the central parts shifted further from the cell membrane (*Figure 5C*), partially covering helices A and B, as well as helix C (*Figure 5A*).

**Figure 6:**
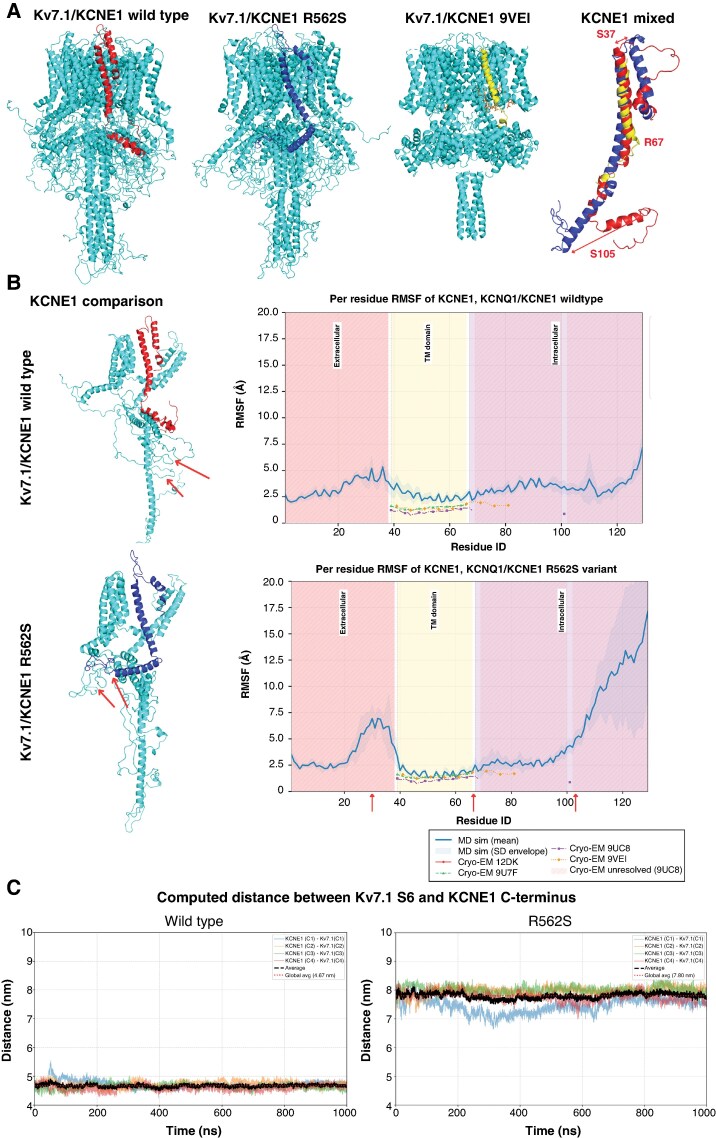
Focus on the position of KCNE1 in the Kv7.1/KCNE1 complex. (*A*) Positioning of KCNE1 in wild type (WT), R562S, and in the experimental structure from cryogenic electron microscopy (cryo-EM) (PDB ID: 9VEI),^26^ with overlapping model of KCNE1 from all three structures (the right panel). Free loops are not visible in the experimental 9VEI structure. The C terminus of KCNE1 in R562S is deflected in the opposite direction compared with wild type (WT). This deflection appears to be associated with the rearrangement of the free loops connecting helices A and B of Kv7.1. (*B*) Comparison of the KCNE1 positioning in the Kv7.1 (one domain model on the left); red arrows show a shift of the free loops. On the right, there are the per-residue root-mean-square fluctuation (RMSF) graphs of WT and R562S variants from the molecular dynamics simulations (compared with available cryo-EM structures) show a large increase in the KCNE1 C-terminal flexibility in the R562S variant; red arrows are pointing to three amino acid residues, S37, R67, and S105 [marked in the right panel of (*A*)]. (*C*) Distances of centres-of mass of the C-terminal part of KCNE1 (S105) and helix S6 (S338 to F340) in Kv7.1 show a large displacement in the R562S variant compared with WT.

The displacement of the free loops also affected the positioning of the modulatory KCNE1 subunit (*Figure 6*). We compared KCNE1 positioning in R562S not only with WT but also with the experimental structures from cryogenic electron microscopy (cryo-EM) with PDB ID: 9VEI (*Figure 6A*), 9UC8, and 9U7F recently published by Cui *et al.*^26^, and newly published structure with PDB ID: 12DK.^27^ The experimental structure, however, does not contain the flexible loop regions. Hence, they were not suitable for comparison with both flexible N and C termini. While the KCNE1 N terminus (S37) and the second binding site for PIP2 (R67) exhibited minor fluctuations across all simulated structures, a pronounced distal displacement of the KCNE1 C terminus (serine 105, S105, and further) was observed in R562S (*Figure* *6A* and *B*). The C-terminal displacement of KCNE1 in R562S (*Figure 6A*) appeared to be driven by a rearrangement of the free loops connecting helices A and B of Kv7.1 (see arrows in *Figure 6B*), as also evident from the change of the position and flexibility of the free loops region between helices A and B around F423 (*Figure* *5B* and *C*).

Confirmation of the shift of the KCNE1 C terminus in R562S was performed *via* RMSF analysis of the WT and R562S MD–refined structures (*Figure 6B*, right panels). An increase in flexibility is evident in the C-terminal region as well as in the amino acid sequence region 20–40. We were unable to fully align these two regions with the experimental cryo-EM structures because they are truncated at these locations, just outside the membrane, and therefore not captured. Our MD–refined structures thus fill this gap and show the likely location of the KCNE1 C terminus in the region of helices A and B of Kv7.1.

As illustrated in *Figure 6C*, an increased distance was apparent between residues S338 to F340 of the transmembrane segment 6 (S6) of Kv7.1, a place essential for the proper interaction of Kv7.1 and KCNE1,^28^ and the C terminus of KCNE1, which was displaced in R562S compared with WT (*Figure 6A*, right panel). This displacement and, consequently, the altered interaction between Kv7.1 and KCNE1 might underlie the impaired response of the R562S variant to β-adrenergic stimulation (for details, see ‘Impaired Reactivity of R562S Channels to β-Adrenergic Stimulation’).

### Impact of R562S variant on cardiac cell function: in silico modelling using the human ventricular cell model

To investigate the impact of the R562S variant on cellular arrhythmogenesis, we incorporated the variant-induced alterations in *I*_Ks_ properties into our model of a human ventricular cardiomyocyte.^19^ Both this variant-specific heterozygous (WT/R562S) model and the corresponding WT model were then run under pro-arrhythmogenic conditions involving β-adrenergic stimulation mediated by 1 μM isoproterenol,^19^ and an associated increase in the stimulation rate from 1 to 2 Hz.

**Figure 7:**
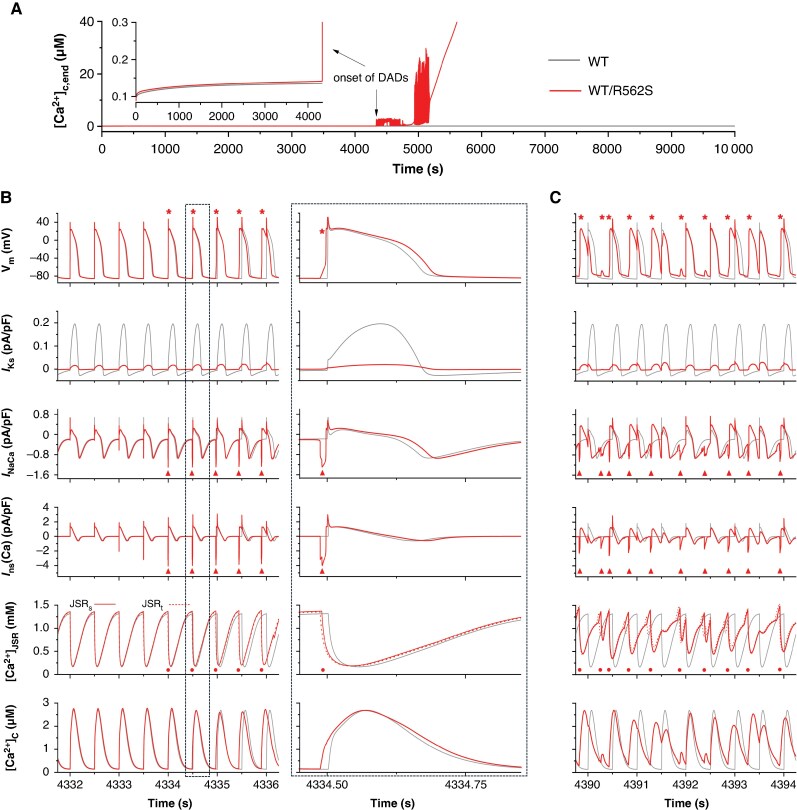
Impact of the wild-type (WT)/R562S variant on the initiation of irregular action potentials (APs) and delayed afterdepolarizations (DADs) during β-adrenergic stimulation associated with increased stimulation frequency from 1 to 2 Hz (extracellular Ca^2+^ concentration 1.82 mM). (*A*) Cytosolic Ca^2+^ concentration at the end of each stimulation cycle ([Ca^2+^]_c,end_). Irregular activity started at 4334 s after initiation of the simulation in the WT/R562S variant. (*B*) The onset of irregular APs (marked by red asterisks) triggered by the activation of inward currents through the Na^+^/Ca^2+^ exchanger *I*_NaCa_ and non-specific Ca^2+^ channel *I*_ns(Ca)_ (red triangles) resulting from irregular Ca^2+^ release from the junctional sarcoplasmic reticulum (red circles) and the consequent development of cytosolic Ca^2+^ transients under intracellular Ca^2+^ overload induced by dysfunctional *I*_Ks_ channels. Note the difference in *I*_Ks_ between WT and WT/R562S. For a detailed view of one of the first DAD-triggered APs (the one marked with a rectangle), see the right panel of (*B*). (*C*) Advanced irregular activity in the model cell with the WT/R562S variant, starting ∼60 s after the onset of the arrhythmia.

When simulations were performed under standard bulk extracellular ion concentrations ([Na^+^]_b_ = 140 mM, [K^+^]_b_ = 5.4 mM, and [Ca^2+^]_b_ = 1.8 mM), no arrhythmogenic activity was observed in both WT and WT/R562S models (not illustrated). However, when [Ca^2+^]_b_ was slightly increased to 1.82 mM (to increase the susceptibility of the model for detecting potential arrhythmogenic effects under β-adrenergic stimulation), the WT/R562S model began to exhibit irregular electrical activity at 4334 s after the start of β-adrenergic stimulation, whereas the WT model remained stable throughout the entire simulated interval of 10 000 s (*Figure 7A*). This difference resulted from a higher intracellular Ca^2+^ load in the WT/R562S model, caused by slower repolarization due to a reduced *I*_Ks_. Consequently, the intracellular Ca^2+^ gradually accumulated until spontaneous Ca^2+^ release events from the junctional sarcoplasmic reticulum occurred (red circles in *Figure* *7B* and *C*). These spontaneous Ca^2+^ releases generated inward depolarizing currents mediated by the Na^+^-Ca^2+^ exchanger current *I*_NaCa_ and non-specific Ca^2+^-activated current *I*_ns(Ca)_ (red triangles in *Figure* *7B* and *C*), leading to delayed afterdepolarizations (DADs). At the onset of arrhythmogenic activity, DADs developed near the end of the stimulation cycle and reached the threshold for triggering full action potentials (red asterisks in *Figure 7B*). As intracellular Ca^2+^ overload further increased, spontaneous Ca^2+^ release events occurred progressively earlier after regular stimulation, shortening the latency between paced action potentials and DAD-triggered depolarizations. Consequently, more complex irregular electrical activity developed, consisting of both triggered action potentials and subthreshold DADs (*Figure 7C*).

**Figure 8:**
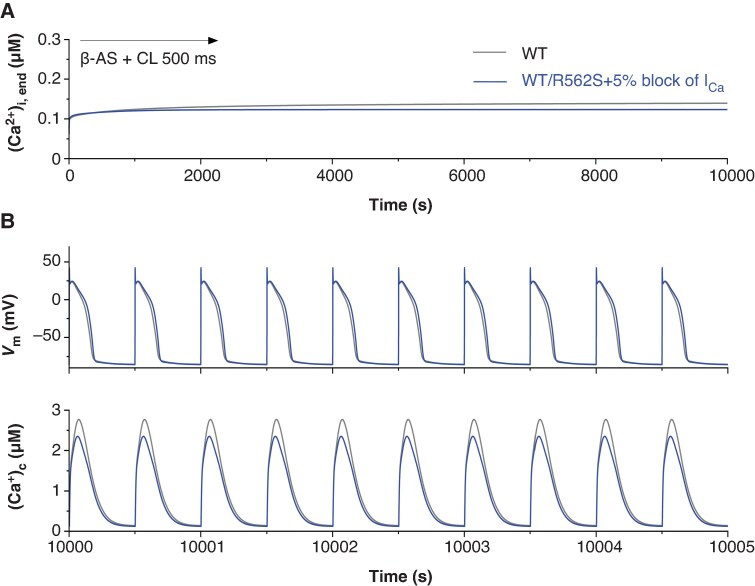
Prevention of the pro-arrhythmic action of wild-type (WT)/R562S channels under β-adrenergic stimulation at a cycle length (CL) of 500 ms (set at 0 s) by a 5% inhibition of the cardiac L-type calcium current (*I*_Ca_). (*A*) Cytosolic [Ca^2+^] at the end of stimulation cycles showing no signs of delayed afterdepolarization (DAD) origin (compare with *Figure 7A*). (*B*) Action potentials (APs) and cytosolic Ca^2+^ transients (CaT). Action potentials were only slightly prolonged in the WT/R562S model under *I*_Ca_ inhibition (by ∼5% at 90% repolarization; upper panel). The peak amplitude of CaT was reduced (by ∼15%; lower panel).

Thus, our simulations suggest that the R562S-Kv7.1 variant may promote arrhythmogenesis under conditions of Ca^2+^ overload by enhancing the susceptibility of cardiomyocytes to DAD-mediated triggered activity. This effect is attributable to a combination of reduced repolarization reserve and elevated intracellular Ca^2+^, which together facilitate spontaneous Ca^2+^ release and the subsequent activation of depolarizing currents. These findings may provide a mechanistic explanation for the pro-arrhythmic potential of R562S in heterozygous carriers *in vivo*, particularly under β-adrenergic stimulation. Importantly, the pro-arrhythmic action of WT/R562S channels under β-adrenergic stimulation was prevented by a 5% inhibition of the L-type Ca^2+^ current (*I*_Ca_) applied before the onset of irregular excitations (*Figure 8*). The developed arrhythmia may be interrupted either by *I*_Ca_ inhibition (50%) or by complete β-blockade, both accompanied by a decrease in the stimulation rate back to 1 Hz (see Supplementary material online, *Figures S8* and *S9*, respectively).

## Discussion

This is the first study reporting clinical manifestation of LQTS in heterozygous carriers of the R562S variant in the Kv7.1 protein, suggesting a possible founder character of this variant in Central Europe, and demonstrating impaired reactivity of both homo- and heterozygous R562S channels to β-adrenergic stimulation, a key physiological regulation of *I*_Ks_ function. Simulations in a human ventricular cell model showed that the decreased *I*_Ks_ promotes arrhythmogenesis *via* DADs during β-adrenergic stimulation. Structural protein modelling suggested that the blunted β-adrenergic response in R562S might be associated with altered positioning of the modulatory KCNE1 subunit.

### *I*_Ks_ channel dysfunction induced by the R562S-Kv7.1 variant under basic conditions

The R562S variant investigated in this study is localized in a highly conserved region within the Kv7.1 C terminus,^9^ namely in helix C with the coiled-coil structure. This region is far from the membrane, but it encompasses a cluster of basic residues (i.e. arginine and lysine residues) that stabilizes the Kv7.1 assembly by interactions between individual subunits within the coiled-coil stem and allosteric interactions *via* the free loops through electrostatic interactions.^29^ As previously demonstrated by Liu *et al.*^10^ using a PIP2 pulldown assay, the C terminus of R562S binds to immobilized PIP2 with lower affinity than the WT C terminus, indicating reduced PIP2-binding capacity associated with R562S. Similar findings have been reported for other helix C variants (e.g. K557E, R555C).^29^

The C terminus of Kv7.1 is also important for the proper trafficking of *I*_Ks_ channels.^8^ The trafficking was not altered by the R562S-Kv7.1 variant (*Figure 2* in the present study, as well as figure 3 in Liu *et al*.^10^).

Taken together, these findings suggest that R562S preserves normal channel trafficking but impairs voltage-dependent activation under basal conditions, most likely by allosteric disruption of PIP2 interactions after reorganization due to destabilization of helix C interactions. Since our models do not contain PIP2 moieties, we cannot measure it directly from the MD simulations.

### Impaired reactivity of R562S channels to β-adrenergic stimulation

Several Kv7.1 variants have been shown to alter the reactivity of *I*_Ks_ to β-adrenergic stimulation (for a review, see Policarová *et al.*^30^). Interestingly, not all C-terminal variants that affect *I*_Ks_ under basal conditions also exhibit impaired reactivity to β-adrenergic stimulation. For example, the previously characterized G589D variant, located in helix D, thus in a proximity to R562S, is associated with LQT1 and also resulted in absent β-adrenergic accumulation of *I*_Ks_.^24,25^ In contrast, other investigated near variants in helix C, namely R555C^31,32^ and K557E,^5^ or slightly more distant A590T^33^ did not affect this regulation of *I*_Ks_. Hence, R562S is the first variant with impaired β-adrenergic response located in helix C of Kv7.1.

The mechanism of the missing responsiveness to β-adrenergic stimulation varies among Kv7.1 variants described so far. In G589D variant, located at the Kv7.1 C terminus near our R562S variant, as mentioned above, an altered interaction between Kv7.1 and the anchoring protein Yotiao was documented, which disabled the response and proved position G589 as the binding site for Yotiao.^24^ Considering the proximity of R562S and G589D, this might be a potential mechanism in our case as well. The other well-known Kv7.1 variant sharing impaired responsiveness to β-adrenergic stimulation, A341V, is located in the S6 transmembrane segment. Under basal conditions, this variant showed a reduced *I*_Ks_,^25^ which was explained by a disrupted coupling between the voltage-sensitive domain and the pore of the Kv7.1 channel.^2^ The mechanism underlying the impaired PKA responsiveness of A341V appears to be different in contrast to G589D. As experimentally proved, A341V preserved the binding of Yotiao, and the phosphorylated channel showed changes typical for β-adrenergic response, but the phosphorylation of S27 was significantly reduced, which suppressed reactivity to β-adrenergic stimulation.^25^ The A341V variant was shown to considerably increase the arrhythmic risk in contrast to A341-neighbouring variants and other missense Kv7.1 variants tested by Schwartz *et al*.^34^

Structural protein modelling with MD simulations may help to explain the missing β-adrenergic response in R562S. R562 is located on the edge of helix C (*Figure 5A*). We observed that the flexible loops between helices A and B (see the position F423 marked by an asterisk in *Figure 5B*) are most affected by the substitution of arginine with serine at position 562, likely due to the loss of a stabilizing interaction between R562 and surrounding amino acid residues. Arginine is positively charged and a multiple hydrogen-bond donor. It can form multiple electrostatic interactions, and its long side chain enables it to extend across structural elements.^35^ This supports the hypothesis that R562 is the stabilizing amino acid for the free loops in the vicinity of the pore. Without this interaction, the free loops became more flexible and shifted further from the cell membrane (*Figure 5C*), namely to the region, which is normally occupied by the C terminus of KCNE1 in WT (*Figure 6A*). An intact C terminus of KCNE1 seems to be critical for modulating Kv7.1^28^ and, importantly, for its proper β-adrenergic response.^36^ Dvir *et al.*^29^ showed that Kv7.1 with the deletion variant Δ109–129 of the distal C terminus of KCNE1 exhibited an impaired β-adrenergic response. In our WT simulation, the C terminus of KCNE1 points correctly towards A-helix and S6 of Kv7.1 (*Figure 6C*). In contrast, the C terminus of KCNE1 is displaced in the R562S variant, as evidenced by the different positions of S105 of KCNE1 (*Figure* *6A* and *C*), and appears more flexible (*Figure 6B*). We therefore hypothesize that the altered position of the KCNE1 C terminus may be responsible for the impaired β-adrenergic response observed in the R562S variant. This feature appears to be unique to R562S, as the nearly located K557E variant preserves β-adrenergic response^5^ despite also being located within helix C and impairing PIP2 binding.^29^

The results of the *in silico* analyses provide only limited insight into the underlying mechanism, as they cannot fully compensate for the lack of experimental data. The performed 1-μs MD simulations in the membrane, although computationally intensive, still encounter the limits of conformational sampling and system simplification. The system used a simple lipid model with the absence of PIP2, and we ran the simulations without any other protein partners of Kv7.1, such as PKA, calmodulin, or Yotiao. Nevertheless, our models correctly captured the published cryo-EM structures (*Figures 5* and *6*), which, however, were limited only to the more rigid parts of the proteins.

### Genotype–phenotype correlation

The amino acid sequence of helices C and D is highly conserved throughout evolution, suggesting limited tolerance for changes in this region.^10^  *I*_Ks_ plays a crucial role in accelerating repolarization during β-adrenergic stimulation. It is therefore not surprising that Kv7.1 variants impairing *I*_Ks_ augmentation under β-adrenergic stimulation exhibit high clinical penetrance, as observed for mutations interfering with the PKA phosphorylation axis.^2^ Notably, the same authors reported high clinical penetrance among variants located within helices C and D, implying that at least some of these variants must disrupt the physiological role of Kv7.1 in mediating β-adrenergic augmentation of *I*_Ks_.^2^

The arrhythmogenic consequences of QTc prolongation are well established.^37^ In LQT1, arrhythmic events are typically triggered by situations requiring an appropriate β-adrenergic response, such as physical exertion.^38^ In R562S carriers, QTc prolongation induced by exercise was significantly higher than in their healthy relatives (*Figure 1F*). This demonstrates the reduced ability of the heterozygous R562S *I*_Ks_ channels to adapt repolarization to β-adrenergic stimulation. As predicted by simulations in the human ventricular cell model, arrhythmogenesis in R562S carriers under conditions of β-adrenergic stimulation may be promoted *via* Ca^2+^ overload and consequently increased susceptibility to DADs (*Figure 7*). In 2021, we suggested the antiarrhythmic effect of preventive 5% *I*_Ca_ inhibition in our previously published variant T309I in the Kv7.1 protein.^19^ Later on, preventing intracellular Ca^2+^ overload through the use of Ca^2+^ channel blockers, such as verapamil, was represented as an emerging therapeutic strategy in LQTS.^37,39^ Its efficacy was also apparent in *in silico* simulations of arrhythmogenesis in the R562S variant (*Figure 8*; see Supplementary material online, Figure  *S8*). The slight *I*_Ca_ inhibition might be beneficial, namely for patients who do not respond to the β-blocking treatment properly or do not tolerate it well.^40^ Nevertheless, β-blockers remain the cornerstone of therapy because β-blockade (at least complete) can effectively prevent the pro-arrhythmic Ca^2+^ overload and consequent DADs under β-adrenergic stimulation (no arrhythmias were observed in the WT/R562S model during *in silico* simulations under control conditions). Moreover, even developed arrhythmia may be interrupted by complete β-blockade (see Supplementary material online, Figure  *S9*). This corresponds to the simulation study by Saucerman *et al.*,^41^ who demonstrated that QTc prolongation, transient afterdepolarizations, an increase in transmural dispersion of repolarization, and T-wave abnormalities could be observed in their G589D ventricular model during β-adrenergic stimulation, but not in its absence.

In the present study, we provide a potential structural explanation for how the C-helix variant R562S may interfere with the PKA phosphorylation axis, thereby leading to prolonged QTc intervals in all examined carriers during physical exercise. Accordingly, the clinical penetrance in our cohort appears to be high, despite all individuals being heterozygous carriers.

## Conclusions

Here, we first report clinical signs of LQTS in heterozygous carriers of the R562S-Kv7.1 variant, which was suggested to be a founder variant in Central Europe. R562S is the first variant in helix C of the Kv7.1 C terminus showing impaired reactivity to β-adrenergic stimulation, a key physiological regulation of *I*_Ks_ channel function. As suggested by the performed MD simulations, the impaired β-adrenergic response of R562S likely results from a mispositioning of the C terminus of the modulatory KCNE1 subunit.

## Supplementary Material

euag156_Supplementary_Data

## Data Availability

The relevant data are available from the authors upon reasonable request.
