# Supplementary material for: C-terminal long-QT type 1 R562S-Kv7.1 variant, the first variant in helix C impairing β-adrenergic response of the slow delayed rectifier K+ channel
Source: Europace. 2026 Jun 18;28(7):euag156. doi: 10.1093/europace/euag156 (PMC13347974; doi:10.1093/europace/euag156)
Supplement: euag156_Supplementary_Data [file euag156_supplementary_data.zip › Kral_et_al_Supplementary_Material_final.pdf]

C-terminal long-QT type 1 R562S-Kv7.1 variant, the first variant in helix C impairing β-adrenergic response of the slow delayed rectifier K^+ channel

SUPPLEMENTARY MATERIAL

Martin Král, Olga Švecová, Roman Kula, Nina Kadášová, Jindřich Lněnička, Iva Synková, Dominika Traj, Larisa Chmelikova, Michal Pásek†, Jan Hošek, Katarzyna Anna Radaszkiewicz, Irena Andršová, Pavel Vít, Karel Berka, Tomáš Novotný, Markéta Bébarová

† in memoriam of Assoc. Prof. Michal Pásek

Correspondence to:

Assoc. Prof. Markéta Bébarová, M.D., Ph.D.

Department of Physiology
Faculty of Medicine
Masaryk University
Kamenice 5
625 00 Brno
Czech Republic

tel. +420-549493147, mbebar@med.muni.cz

Supplementary Methods

Clinical diagnostics

Patients with suspected LQTS are regularly investigated at the Department of Internal Medicine and Cardiology, and at the Department of Paediatrics (both at the University Hospital Brno and Faculty of Medicine, Masaryk University, Brno, Czech Republic). The diagnosis is established according to ESC Guidelines (Priori *et al.* 2015). The study conformed to the principles outlined in the Declaration of Helsinki. All participants signed a written consent form prior to their inclusion in the study. In the case of participants under the age of 18 years, the written informed consent was obtained from a parent and/or legal guardian. The study was approved by the Multicenter Ethical Committee, University Hospital Brno (Brno, Czech Republic).

All individuals included in this study underwent clinical examination and bicycle ergometry to obtain ECG traces at different adrenergic states. A 12-lead ECG with Mason-Likar modification was used. The initial stress was set to 0.5 W/kg and increased by 0.5 W/kg every three minutes to achieve a heart rate higher than the submaximal value for age and sex.

All ECGs were recorded as paper printings at the speed of 50 mm/s and voltage of 20 mm/mV, and QT and RR intervals were measured manually for the periods of rest and in the fourth minute of the recovery period of the exercise test. In most cases, the QT interval was measured in the lead V5; the other leads were used only when the end of the T wave could not be discriminated in this lead. If the end of the T wave was not clearly visible, the threshold method was used. The QT intervals were corrected for the respective heart rate using the Bazett's formula: $QTc = QT/\sqrt{RR}$ (both intervals were measured in seconds).

Genetic testing

Between 2000 and 2018, 132 unrelated index cases with LQTS susceptibility were

examined at the Department of Medical Genetics (University Hospital Brno and Faculty of Medicine, Masaryk University, Brno, Czech Republic). Informed consents and peripheral blood samples were collected from patients. DNA was extracted by standard molecular techniques. Molecular analysis of LQTS-associated genes, including the *KCNQ1* gene, was performed according to current practises for molecular genetics diagnostics. The classical method (multiplex PCR/SSCP analysis of 3 LQTS major genes) was followed by Sanger sequencing on ABI 3100 Genetic Analyser (*Applied Biosystems*™, Foster City, CA, USA). Primers for the screening of entire coding regions of genes *KCNQ1, KCNH2* and *SCN5A* were designed with Primer-BLAST tool (Ye *et al.* 2012). Direct Sanger sequencing was then replaced with massive parallel sequencing (MPS) of 5 LQTS-related genes (*KCNQ1*, *KCNH2*, *SCN5A*, *KCNE1*, *KCNE2*) on GS Junior (*Roche*, Basel, Switzerland). The sequencing library in this case was prepared with a hybridization capture-based target enrichment method using NimbleGen SeqCap EZ Choice Library Kit (*Roche*, Basel, Switzerland). Since 2016, MPS of 13 LQTS-associated genes (*KCNQ1*, *KCNH2*, *SCN5A*, *AKAP9*, *CACNA1C*, *CALM1*, *CALM2*, *CAV3*, *KCNE1*, *KCNE2*, *KCNJ5*, *SCN4B*, *SNTA1*) has been performed on MiSeq (*Illumina*, San Diego, CA, USA). Two types of sequencing library have been used: 1) Amplicon library prepared with commercial kit TruSeq Custom Amplicon Kit (*Illumina*, San Diego, CA, USA); and 2) Target enriched library prepared with hybridization capture-based method using KAPA HyperPlus Kit with SeqCap EZ Choice Library Kit (*Roche*, Basel, Switzerland). The procedure has been performed as recommended by the manufacturer. Genetic counselling and testing of first-degree relatives have been offered to patients at risk.

Prediction of the channel dysfunction

The tools MutationTaster and FATHMM (Shihab *et al.* 2014) were used to predict the possible clinical impact of the identified sequence variant. Conservation of the affected amino

acid position was assessed using the Likelihood Ratio Test (LRT) and MutationAssessor. Allele frequency of the substitution was determined using online databases ExAC (Lek *et al.* 2016) and GnomAD (Karczewski *et al.* 2020).

Haplotype analysis

For the haplotype analysis, 9 STR (short tandem repeats) markers spanning the ~11.9-Mb region of chromosome 11 (including the *KCNQ1* gene) were chosen from the University of California, Santa Cruz (UCSC) Genome Browser: D11S1363, D11S922, D11S4046, D11S4088, D11S4146, D11S1760, D11S1338, D11S4149, D11S4116 (Suppl. Tab. S1). Multiplex PCR with fluorescently labelled primers and fragment analysis with capillary electrophoresis were performed on SeqStudio Genetic Analyzer (*Applied Biosystems*™, Foster City, CA, USA). The haplotype linked to the mutation was identified by studying segregation in families.

Population allele frequency analysis was performed after identifying a common STR allele in the marker D11S4088 in all of the affected individuals. The control group was formed by 52 unrelated patients examined at the Department of Clinical Genetics, Faculty Hospital Brno, with a signed informed consent from each patient agreeing that their DNA samples could be used for clinical research. STR alleles (104) were amplified with fluorescently labelled primers and analysed with capillary electrophoresis.

Suppl. Tab. S1: Analysed STR markers and their distance to the mutation

marker	distance (bp)
D11S1363	-1735912
D11S922	-1193015
D11S4046	-834581
D11S4088	-42990
c.1686C>G	0
D11S1923	826640
D11S4146	1330967
D11S1760	2973215
D11S1338	3576790
D11S4149	6718788
D11S4116	10539477
D11S902	15077320

(UCSC Genome Browser; www.genome.ucsc.edu/)

Biophysical analysis

Wild-type (WT) human *KCNQ1* in a pIRES2-eGFP vector and WT human *KCNE1* in a pKB-CMV vector were kindly provided by Prof. Paul G.A. Volders, MD, PhD (Maastricht University, Maastricht, Netherlands). *Yotiao* in a pGW1 vector was the kind gift of Prof. Robert R. Kass (Columbia University, New York, USA).

Plasmid DNA was cloned into chemically competent *Escherichia coli* cells from NovaBlue Singles™ Competent Cells (*Novagen*, Madison, WI, USA) by the heat-shock technique according to the manufacturer's manual. After cultivation, plasmids were isolated from bacterial cells using endotoxin-free QIAprep Spin Miniprep Kit (*Qiagen*, Hilden, Germany). The quantity and purity of isolated plasmids were measured spectrophotometrically by BioPhotometer (*Eppendorf*, Hamburg, Germany).

The mutation c.1686G>C in the human *KCNQ1* (p.R562S) was generated by site-directed mutagenesis using QuikChange II XL Site-Directed Mutagenesis Kit (*Agilent Technologies*, Cedar Creek, TX, USA) with the following primers:

5'- GGA CTG GTC CAG GCT CCT CTG CAG C-3'

5'- GCT GCA GAG GAG CCT GGA CCA GTC C-3'

The presence of the mutation in *KCNQ1* was verified by sequencing performed by the SEQme company (Dobříš, Czech Republic).

TransFast Transfection Reagent (*Promega*, Madison, WI, USA) was used for transfection of the plasmids (*KCNQ1*, *KCNE1*, and *Yotiao* in the molar ratio 1:2:4, total amount of DNA was 1 µg and the ratio of DNA to transfection agent was 1:1.5) into Chinese hamster ovary (CHO) cells that were cultured at 37°C / 5% CO_2 in Ham's F-12 medium supplemented with 10% foetal calf serum and 0.005% gentamycin (*Sigma-Aldrich*, St. Louis, MO, USA). *KCNQ1* was transfected in one of three ways: 1) WT variant alone (WT); 2) R562S variant alone (R562S); 3) both the WT and R562S variants cotransfected in the ratio 1:1 (WT/R562S) to mimic the heterozygous state in the mutation carriers.

Measurements were performed ~24 h after the transfection by the whole cell patch clamp technique in the voltage clamp mode at 37°C. The patch pipettes were pulled from borosilicate glass capillary tubes and heat-polished on a programmable horizontal puller (*Zeitz-Instruments Vertriebs GmbH*, Martinsried, Germany). The resistance of the filled glass electrodes was below 2.5 MΩ to keep the access resistance as low as possible. For the generation of experimental protocols and data acquisition, the Axopatch 200A equipment and pCLAMP 9.2 software (*Molecular Devices*, San José, CA, USA) were used. The series resistance was compensated up to 60%. The measured ionic currents were digitally sampled at 2 kHz (after low-pass filtering at 5 kHz) and stored on the hard disc. For the experimental protocols, please see Results; if not mentioned, the stimulation frequency was 0.08 Hz.

Tyrode solution of the following composition was used to perfuse the measured cells (in mmol/L): NaCl 132, KCl 4.8, $CaCl_2$ 1.8, $MgCl_2$ 1.2, HEPES 10, glucose 5 (pH was adjusted to 7.4 with NaOH). The patch electrode filling solution contained (in mmol/L): K-aspartate 110,

K_2ATP 5, $CaCl_2$ 1, $MgCl_2$ 1, EGTA 11, HEPES 10 (pH 7.3 adjusted with KOH). The junction potential was +15 mV. To simulate β-adrenergic stimulation, the pipette solution was supplemented with cyclic adenosine monophosphate (cAMP, 200 μmol/L) and an inhibitor of serine/threonine phosphatases okadaic acid (OA, 0.2 μmol/L) in some experiments. The stock solutions were kept frozen and the chemicals were added to the pipette solution before the measurements were started. The pipette solution was subsequently kept in the fridge, lying on ice, before being filled to the measuring micropipette. Unless otherwise indicated, the chemicals were purchased from *Sigma-Aldrich* (Prague, Czech Republic).

The normal data distribution was rejected in the case of cell membrane capacitance by both the Shapiro-Wilk and Anderson-Darling tests (Suppl. Fig. S1), thus, the geometric mean ± SD is stated, based on the recommendation by Kula *et al.* (2020a). The average cell membrane capacitance was comparable in WT, R562S, and WT/R562S I_{Ks} channels (13.5*/1.4, 13.7*/1.6, and 12.9*/1.6 pF, respectively, $n = 21, 28$, and 20; $P > 0.05$, Kruskal-Wallis test with the Dunn´s post-test).

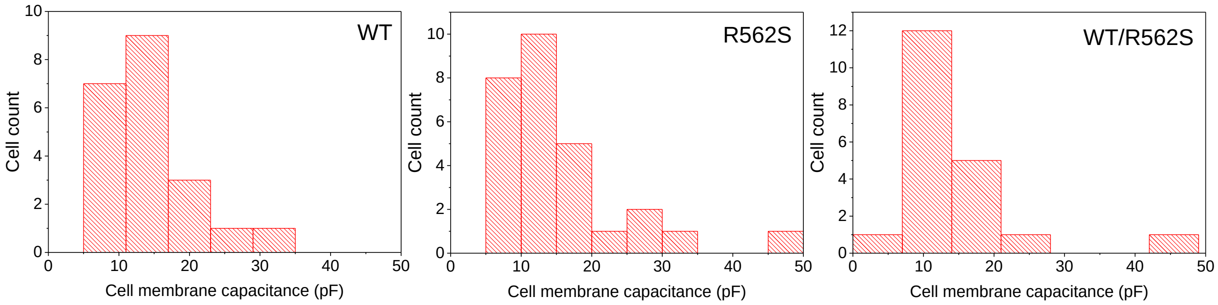

Suppl. Fig. S1: Histograms showing skewed frequency distribution of the cell membrane capacitance in all cell groups.

Considering the missing proportionality between the measured tail current and estimated cell membrane capacitance (Suppl. Fig. S2), conversion of the magnitude of the current to the current density was avoided in this study, as recently recommended by Kula *et al.* (2020b).

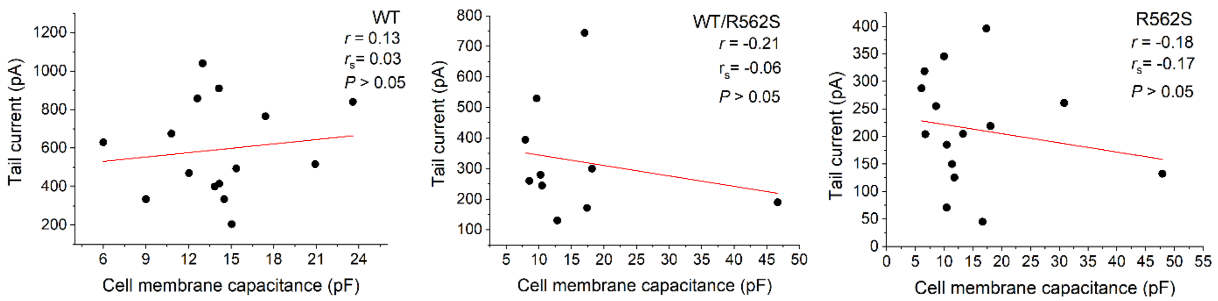

Suppl. Fig. S2: Magnitude of the tail current did not correlate with the cell membrane capacitance in any cell group, thus, conversion of the tail current magnitude to the current density was not performed; *r* and r_s – the Pearson´s and Spearman´s correlation coefficients, respectively. In the WT/R562S and R562S groups, one membrane capacitance value in each dataset fulfilled the 1.5× interquartile range criterion for a statistical outlier (46.5 pF and 48 pF, respectively). However, the absence of proportionality between tail current magnitude and membrane capacitance persisted even after exclusion of these values (WT/R562S: $r = 0.124$, $P = 0.750$, and $r_s = 0.067$, $P = 0.864$; R562S: $r = -0.016$, $P = 0.958$, and $r_s = -0.002$, $P = 0.995$).

For comparison with previously published electrophysiological studies, current amplitudes were additionally normalized to the cell membrane capacitance and expressed as the current density (pA/pF). Suppl. Fig. S3 shows the current density–voltage relationship as a complement to Fig. 3C in the main text. The normalization did not alter the observed differences between groups. Thus, the overall results remained comparable to those obtained using the raw current amplitudes.

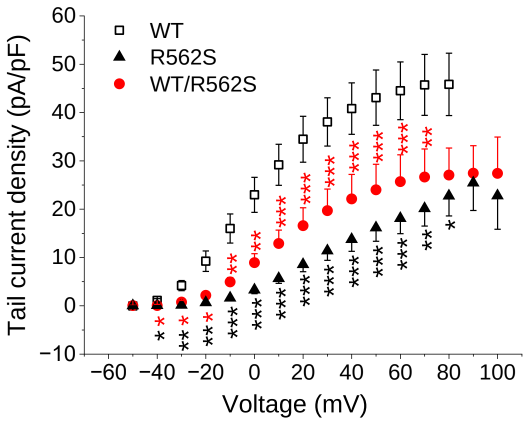

Suppl. Fig. S3: Average current density-voltage relationship in WT, R562S, and WT/R562S channels. Statistical comparison was performed between WT *vs.* R562S and WT *vs.* WT/R562S; *, **, and *** – statistical significance at $P < 0.05$, 0.01, and 0.001, respectively (one-way ANOVA with the Bonferroni post-test).

The voltage dependence of steady-state activation was fitted using the Boltzmann equation: $y = I_{max} / (1 + \exp((V_{1/2} - V) / k))$ to determine the half-maximal activation voltage $V_{1/2}$ and the slope factor k. Time courses of activation and deactivation were fitted with a single exponential function: $y = A \cdot (1 - \exp(-t/\tau))$, where A is the amplitude and τ is the time constant.

Confocal microscopy

WT human *KCNQ1* tagged with GFP at the 3'-terminus or without GFP in a pBK-CMV vector (vector without GFP marker), as well as WT human *KCNE1* in a pBK-CMV vector were kindly provided by Prof. Paul G.A. Volders, MD, PhD (Maastricht University, Maastricht, Netherlands). The mutation c.1686G>C (p.R562S) in the human *KCNQ1* with tagged GFP was generated by the site-directed mutagenesis technique, as described above.

TransFast Transfection Reagent (*Promega*, Madison, WI, USA) was used for transfection of the plasmids (*KCNQ1* and *KCNE1* in the molar ratio 1:2, the total amount of DNA was 1 μg and the ratio of DNA to transfection agent was 1:1.5) into CHO cells. The cells were cultured at 37°C / 5% CO_2 in Ham's F-12 medium supplemented with 10% foetal calf serum and 0.005% gentamycin (*Sigma-Aldrich*, St. Louis, MO, USA) in glass bottom dishes (*Cellvis*, Mountain View, CA, USA) coated with fibronectin (*Sigma-Aldrich*, St. Louis, MO, USA) to enhance cell adhesion. After ~24 h, the CHO cells were transiently transfected in one of three ways: 1) WT variant alone (WT-GFP); 2) R562S variant alone (R562S-GFP); 3) both the WT and R562S variants cotransfected in the ratio 1:1 (WT-GFP/R562S-GFP).

Measurements were performed ~48 h after the transfection. A confocal laser scanning microscope Leica TCS SP8 X (*Leica Microsystems*, Wetzlar, Germany) was used to analyse the subcellular localization of WT, R562S, and WT/R562S I_{Ks} channels. Excitation wavelength was set to 488 nm and emission range to 500-560 nm, corresponding to the GFP spectral properties. The samples were observed using an oil lens 63X objective. The acquired images (at a spatial

resolution of 1024 × 1024 pixels, physical length of 184.52 × 184.52 μm, and bit depth of 8 bit) were averaged three times and exported in the TIFF image format. GFP localization was analysed by relative intensity of line plot profiles.

In silico structural modelling and molecular dynamics simulations

To create the model of I_{Ks} channel using AlphaFold 3[17], amino acid sequences of Kv7.1 and KCNE1 were retrieved from the UniProt database (identifiers P51787 and P15382, respectively; Kv7.1 and KCNE1 in a ratio 1:1)[18,19]. To create a physiological tetramer of Kv7.1/KCNE1, the amino acid chains were repeated four times in the folding process. Tetrameric models from AlphaFold 3 (Abramson *et al.* 2024) were generated using 50 repetitions of either oleic acid or myristic acid to predict the pose of the Kv7.1 channel complex in the membrane. The fatty acids were removed before building production MD systems.

Inputs for production MD simulations were prepared in the CHARMM-GUI interface (Jo *et al.* 2008) with the Membrane Builder (Feng *et al.* 2023). The channel was then embedded in a lipid bilayer composed of an equimolar mixture of palmitoyl-oleoyl phosphatidylcholine and palmitoyl-oleoyl phosphatidylethanolamine (orientation and position were assigned by CHARMM-GUI itself and were manually checked against the predicted pose from AlphaFold 3). Systems were built in a rectangular simulation box. The box size was set to at least 2 nm from the protein to ensure that it would not interact with itself through periodic boundary conditions. This resulted in systems varying in size from 19 to 21 nm along the x and y axes and from 18 to 25 nm along the z axis. The lipid bilayer was always built after determining the size along the x- and y-axes. Systems were then minimised and equilibrated with an increasing time step and concurrent release of position restraints on both lipid heads and protein.

Analyses were performed using the Gromacs analysis suite of programs – specifically, gmx rmsf and gmx mindist. Visualisations of models were performed in PyMol 3.1.0.

Modification of the human ventricular cell model

The simulations were performed using our previously published model by Synková *et al.* (2021). To describe the new experimental data characterising the steady-state activation of I_{Ks} under control (WT) conditions and under the effect of the WT/R562S mutation in the model more accurately than allowed by the Boltzmann equation, we used the following formulations:

$$I_{WT} = \frac{1}{(1+e^{(-(V+33.22)/20.98)})^4}$$

$$I_{WT/R562S} = \frac{1}{(1+e^{(-(V+26.89)/23.99)})^4}.$$

Additionally, to account for the observed reduction of the I_{Ks} current in the presence of the WT/R562S mutation, the maximum conductance (g_{Ks}) was decreased by 50%.

The basic units in which the equations were solved were mV for the membrane potential, mA for membrane currents, mM for ionic concentrations, ml for volumes, and s for the time.

Supplementary Results

Examples of ECG recordings at rest and after exercise

Rest | 4th min of the recovery after exercise

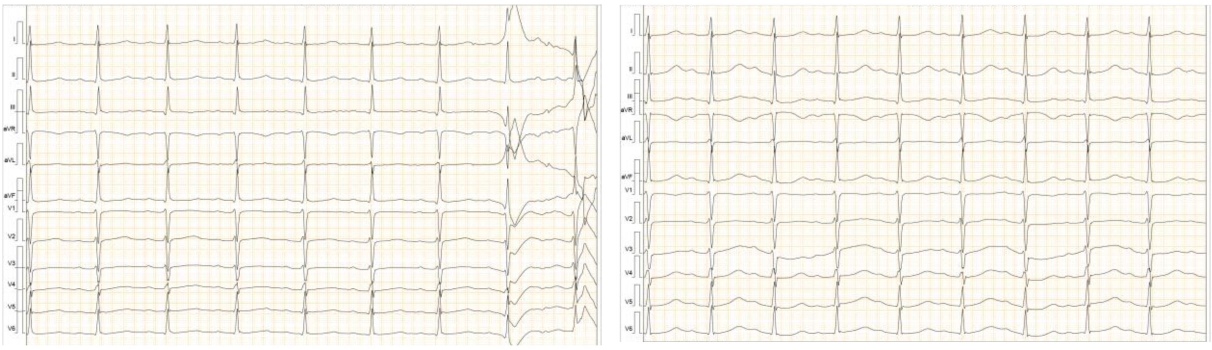

Suppl. Fig. S4: Representative ECG recordings (50 mm/s) of the patient I:3 (see Fig. 1), the proband of Family I, carrying only the R562S-Kv7.1 variant.

Rest | 4th min of the recovery after exercise

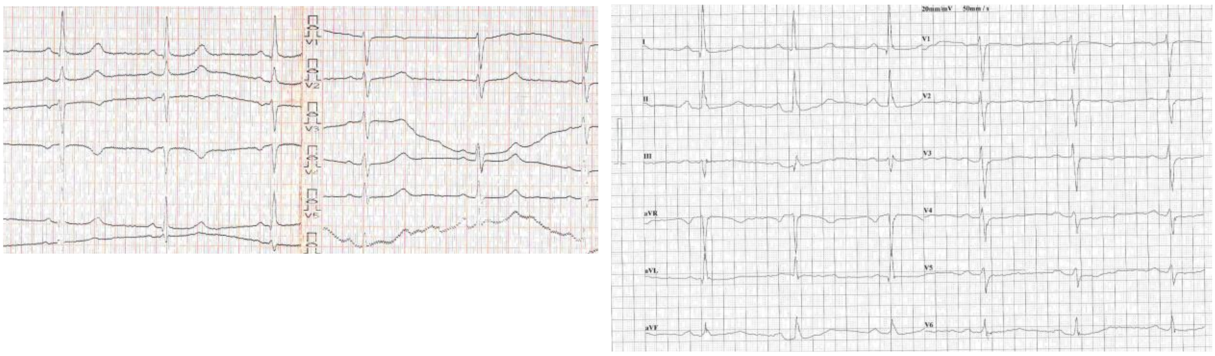

Suppl. Fig. S5: Representative ECG recordings (50 mm/s) of the patient III:2 (see Fig. 1), the mother of the proband of Family III, carrying only the R562S-Kv7.1 variant.

Rest | 4th min of the recovery after exercise

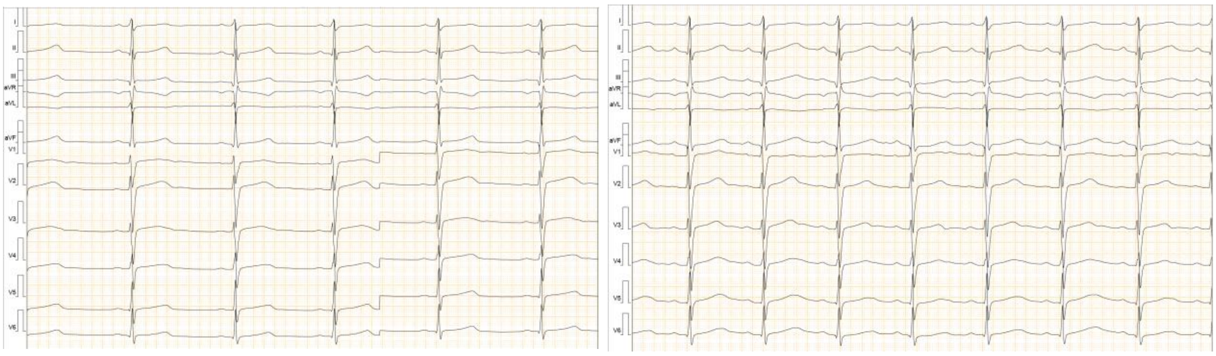

Suppl. Fig. S6: Representative ECG recordings (50 mm/s) of the patient IV:6 (see Fig. 1), the proband of Family IV, carrying the following variants: R562S-Kv7.1, R176W-hERG, and V1359A-RYR2.

Original AlphaFold 3 models of the WT and R562S variants

Suppl. Fig. S7 shows a comparison of the structure of the WT- and R562S-Kv7.1 variants in the tetrameric complex, together with the KCNE1 subunits (Dixit *et al.* 2020).

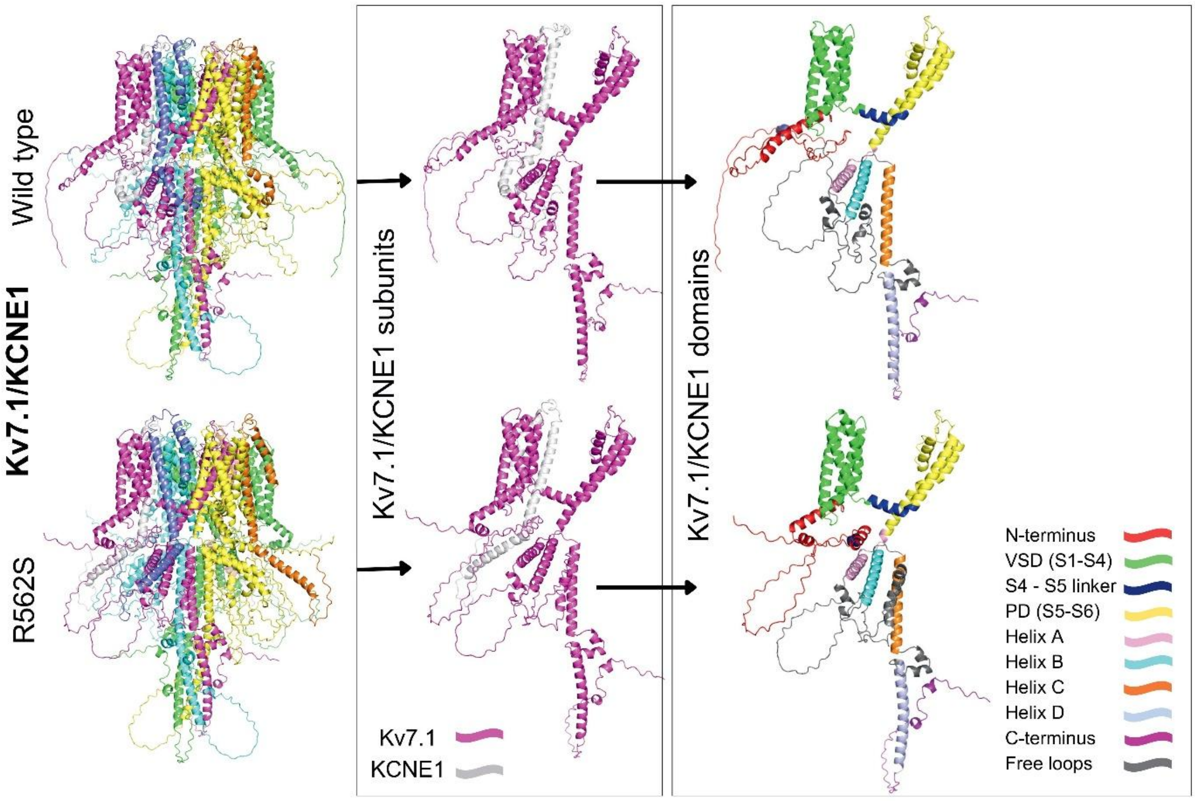

Suppl. Fig. S7: Comparison of the original AlphaFold 3 models of wild type (WT) and R562S variants in complete tetrameric structure and with the modulatory KCNE1 subunits (left), the same in the monomeric structure (middle), and positions of the individual channel domains (right).

Simulation of the antiarrhythmic effect of a partial I_{Ca} inhibition

As shown in Fig. 8 in the main text, the proarrhythmic action of WT/R562S channels under β-adrenergic stimulation (β-AS) was prevented by a 5% inhibition of the L-type calcium current (I_{Ca}) applied before the onset of irregular excitations in the human ventricular cell model. However, this inhibition was not sufficient in the acute state, when arrhythmogenic activity was fully developed (~60 s after the onset of irregular action potentials). In this state, the development of delayed afterdepolarizations (DADs) triggering irregular action potentials

was halted after a ~70 s delay following a 50% inhibition of I_{Ca} and a reduction in stimulation rate from 2 to 1 Hz (Suppl. Fig. S8). The same applies to complete inhibition of β-adrenergic response (Suppl. Fig. S9).

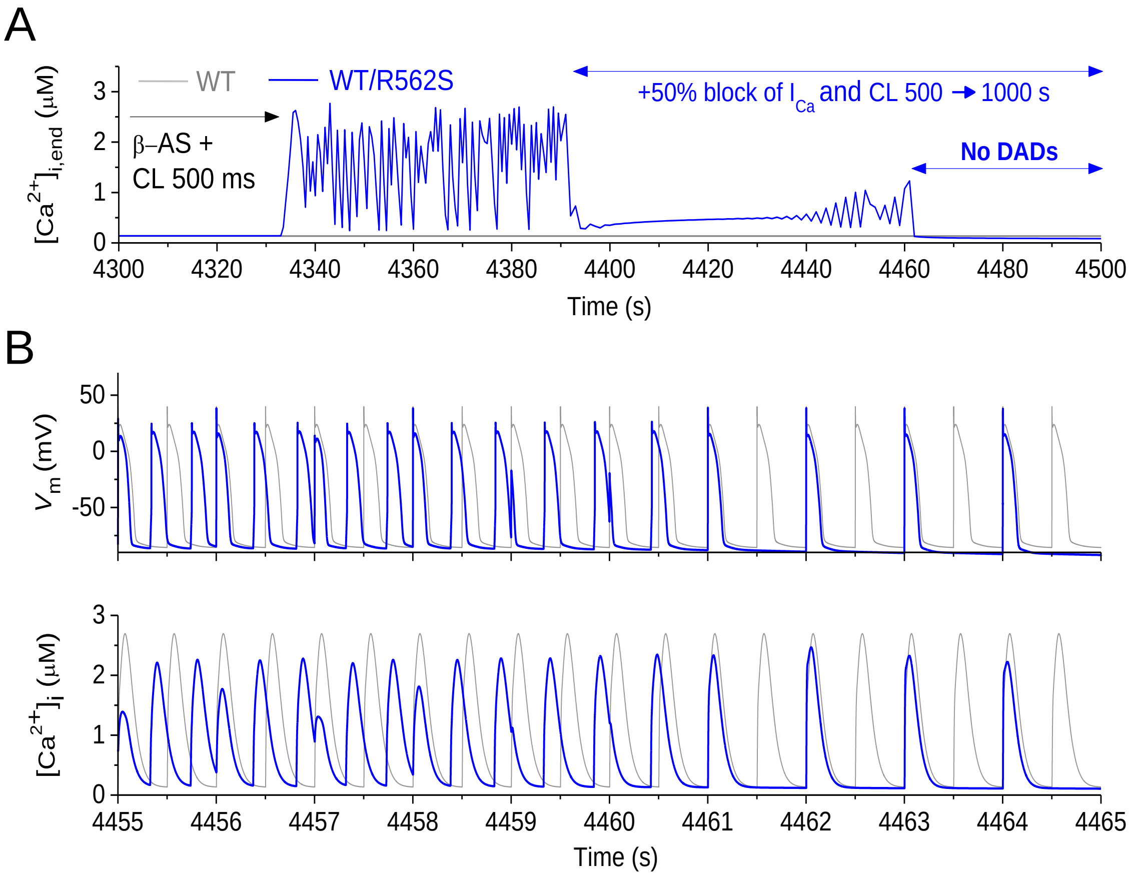

Suppl. Fig. S8: Suppression of the proarrhythmic action of WT/R562S channels under β-AS (set at 0 s) by a 50% inhibition of I_{Ca} and a decrease in the stimulation rate from 2 to 1 Hz. (A) Cytosolic [Ca^{2+}] at the end of stimulation cycles, showing advanced arrhythmogenic activity and its suppression 70 s after the onset of I_{Ca} inhibition (at 4390 s in the WT/R562S model). (B) Corresponding APs and CaT in both WT and WT/R562S models.

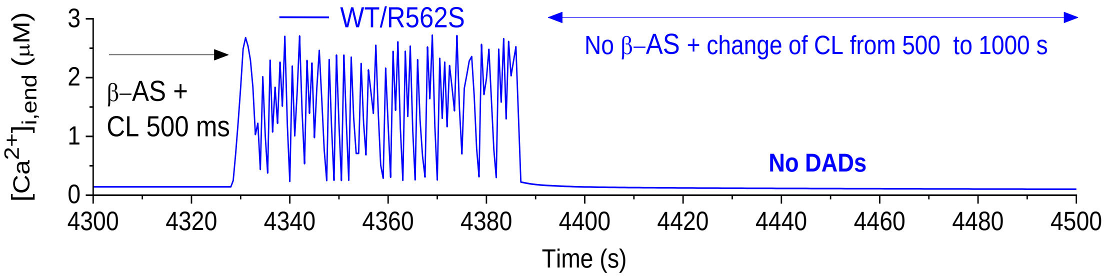

Suppl. Fig. S9: Cytosolic [Ca^{2+}] at the end of stimulation cycles, showing advanced arrhythmogenic activity under β-AS (set at 0 s), which was suppressed by total block of β-AS and a decrease in the stimulation rate from 2 to 1 Hz.

References:

Abramson J, Adler J, Dunger J, *et al.* Accurate structure prediction of biomolecular interactions with AlphaFold 3. Nature 2024;630(8016):493-500.

Dixit G, Dabney-Smith C, Lorigan GA. The membrane protein KCNQ1 potassium ion channel: Functional diversity and current structural insights. Biochim Biophys Acta Biomembr. 2020;1862(5):183148.

Feng S, Park S, Choi YK, *et al.* CHARMM-GUI Membrane Builder: Past, Current, and Future Developments and Applications. J Chem Theory Comput 2023;19(8):2161-2185.

Jo S, Kim T, Iyer VG, *et al.* CHARMM-GUI: a web-based graphical user interface for CHARMM. J Comput Chem 2008;29(11):1859-1865.

Karczewski KJ, Francioli LC, Tiao G, *et al.* The mutational constraint spectrum quantified from variation in 141,456 humans. Nature 2020;581(7809):434-443.

Kula R, Bébarová M, Matejovič P, *et al.* Distribution of data in cellular electrophysiology: Is it always normal? Prog Biophys Mol Biol 2020a;157:11-17.

Kula R, Bébarová M, Matejovič P, *et al.* Current density as routine parameter for description of ionic membrane current: is it always the best option? Prog Biophys Mol Biol 2020b;157:24-32.

Lek M, Karczewski KJ, Minikel EV, *et al.* Analysis of protein-coding genetic variation in 60,706 humans. Nature 2016;536(7616):285-291.

Priori SG, Blomström-Lundqvist C, Mazzanti A, *et al.* 2015 ESC Guidelines for the management of patients with ventricular arrhythmias and the prevention of sudden cardiac death: The Task Force for the Management of Patients with Ventricular Arrhythmias and the Prevention of Sudden Cardiac Death of the European Society of Cardiology (ESC). Endorsed by: Association for European Paediatric and Congenital Cardiology (AEPC). Eur Heart J 2015;36(41):2793-2867.

Shihab HA, Gough J, Mort M, *et al.* Ranking non-synonymous single nucleotide polymorphisms based on disease concepts. Hum Genomics 2014;8:11.

Synková I, Bébarová M, Andršová I, *et al.* Long-QT founder variant T309I-Kv7.1 with dominant negative pattern may predispose delayed afterdepolarizations under β-adrenergic stimulation. Sci Rep. 2021;11(1):3573. doi: 10.1038/s41598-021-81670-1.

Ye J, Coulouris G, Zaretskaya I, *et al.* Primer-BLAST: a tool to design target-specific primers for polymerase chain reaction. BMC Bioinformatics 2012;13:134.
